# RNA‐DNA Fusomer Fibers With Customizable Physicochemical, Mechanical, and Biological Properties for Next‐Generation Therapeutics

**DOI:** 10.1002/smll.202600078

**Published:** 2026-06-09

**Authors:** Yasmine Radwan, Laura P. Rebolledo, Yelixza I. Avila, Elizabeth Skelly, Lauren Rackley, Julio Navas Hernandez, Renata de Freitas Saito, Laxmi K. Pandey, Hemani Chhabra, Alexander J. Lushnikov, Tatiane Katsue Furuya, Ana Luiza Lomba, Da Shi, Edward Cedrone, Ian Marriott, Morgan R. Chandler, Meni Wanunu, Aleksei Aksimentiev, Alexey V. Krasnoslobodtsev, Roger Chammas, Marina A. Dobrovolskaia, Kirill A. Afonin

**Affiliations:** ^1^ Chemistry and Nanoscale Science Program Department of Chemistry University of North Carolina Charlotte Charlotte North Carolina USA; ^2^ Department of Physics University of Nebraska Omaha Omaha Nebraska USA; ^3^ Center for Translational Research in Oncology (LIM24) Instituto do Câncer do Estado de São Paulo (ICESP) Hospital das Clínicas da Faculdade de Medicina da Universidade de São Paulo (HCFMUSP) São Paulo SP Brazil; ^4^ Comprehensive Center for Precision Oncology Universidade de São Paulo São Paulo SP Brazil; ^5^ Department of Physics Northeastern University Boston Massachusetts USA; ^6^ Beckman Institute for Advanced Science and Technology University of Illinois at Urbana‐Champaign Urbana Illinois USA; ^7^ Nanotechnology Characterization Laboratory Cancer Research Technology Program Frederick National Laboratory for Cancer Research sponsored by the National Cancer Institute Frederick Maryland USA; ^8^ Department of Biological Sciences University of North Carolina at Charlotte Charlotte North Carolina USA; ^9^ Center for Innovation Translational Research and Applications of Nanostructured Systems (CITRANS) University of North Carolina at Charlotte Charlotte North Carolina USA; ^10^ MIMETAS US INC Gaithersburg Maryland USA; ^11^ Department of Physics University of Illinois at Urbana‐Champaign Urbana Illinois USA

**Keywords:** anti‐coagulation, fusomers, immunomodulation, NANPs, new approach methodologies (NAMs), NF‐κB, silver nanoclusters, solid‐state nanopores

## Abstract

We introduce RNA‐DNA fusomers, a new class of chemically synthesized oligonucleotides that combine the versatile properties of RNA and DNA within a single sequence and self‐assemble into higher‐order functional structures via a simple one‐pot annealing reaction. This hybrid platform allows precise customization with therapeutic nucleic acids, offering tunable physicochemical, mechanical, and immunological properties, cost‐effective production, and the capacity to integrate biological functionalities intrinsic to both RNA and DNA. The modular architecture of fusomers enables straightforward optimization for diverse biomedical applications, including gene silencing, anti‐inflammatory therapy, anticoagulation, antibacterial activity, and protein biosensing. We demonstrate efficient delivery and intracellular modulation by fusomers in multiple model systems, including human peripheral blood mononuclear cells isolated from healthy human donors and 3D organ‐on‐a‐chip models. Molecular dynamics simulations further elucidate the structural behavior of fusomers and their intended interactions with protein targets. Collectively, these findings position fusomers as a next‐generation therapeutic platform with broad transformative potential.

## Graphical abstract

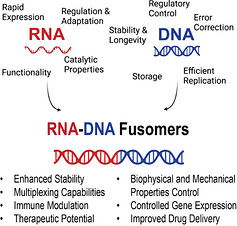

## 1 Introduction

Despite their similarities in chemical composition, RNA and DNA are fundamentally distinct and functionally diverse biopolymers that are essential for all life forms. The uniqueness of each arises from its complementary biological roles and chemical properties. DNA primarily serves as the long‐term repository of genetic information, ensuring its accurate replication and continuity across generations [1]. DNA's double‐stranded (ds) structure and repair mechanisms provide the stability and longevity required for maintaining genomic integrity [2, 3], while serving as a template for RNA synthesis during transcription [4]. In contrast, RNA provides structural flexibility and functional diversity, which are critical for dynamic cellular regulation and the orchestration of major biochemical processes. RNA plays central roles in gene expression, acting as a messenger, translator, regulator, and catalyst [5]. The diverse roles of regulatory RNAs, including microRNAs (miRNAs), small interfering RNAs (siRNAs), riboswitches, and long noncoding RNAs (lncRNAs), further underscore RNA's essential contribution to gene expression and cellular control [5, 6].

The central involvement of DNA and RNA in key biological processes, together with their extraordinary programmability, has made them powerful tools widely applied in both basic research and therapeutic development. To date, more than 40 DNA‐based therapeutics have received FDA approval, including gene therapies [7], gene‐edited cell therapies [8], and chimeric antigen receptor T‐cell (CAR‐T) therapies [9]. For RNA therapeutics [10, 11], more than 20 FDA approved therapies are currently available including antisense oligonucleotides (ASOs), siRNA, mRNA vaccines, and aptamers [12]. These therapeutic nucleic acids (TNAs) have revolutionized the biomedicine landscape due to their selectivity, high efficacy, and cell targeting [13, 14].

The ability to rationally design nucleic acids that self‐assemble with exceptional precision has driven the rise of nucleic acid nanotechnology as an interdisciplinary field for engineering nanoparticles with therapeutic and diagnostic functions [15, 16, 17, 18, 19]. Nucleic acid nanoparticles, or NANPs, represent a unique class of functional biomaterials that are fully customizable to achieve desired physicochemical, mechanical, and biological properties for a broad range of applications. Upon embedding various TNAs, targeting ligands, fluorophores, small molecule drugs, and additional cargos into the NANPs structures [20], their potential use for the treatment of a wide array of diseases has been demonstrated [21, 22, 23]. In addition to functionalization [24], the overall dimensionality [25] and chemical composition [26] of NANPs correlates with their immunostimulatory properties [27]. From extensive studies, 3D RNA‐based NANPs were found to be highly immunostimulatory, whereas DNA‐based and 1D fibrous NANPs were either weakly immunostimulatory or immunoquiescent [25, 28, 29, 30].

Although some NANP design strategies, which rely on the intermolecular assembly of individual strands, allow for the incorporation of both RNA and DNA within the same structure [26, 31], NANPs that rely on intramolecular folding to initiate long‐range RNA interactions [32, 33] cannot tolerate substitutions with DNA strands. This limitation has motivated the development and implementation of the RNA‐DNA fusomer concept, a platform where single sequences integrate both RNA and DNA nucleotides and can self‐assemble into NANPs via RNA‐RNA interactions. This approach enables seamless fusion of the diverse functionalities of both DNA and RNA while preserving the unique structural and long‐range interaction motifs of RNA, thus expanding the versatility and applications of therapeutic nucleic acid nanotechnology.

**FIGURE 1:**
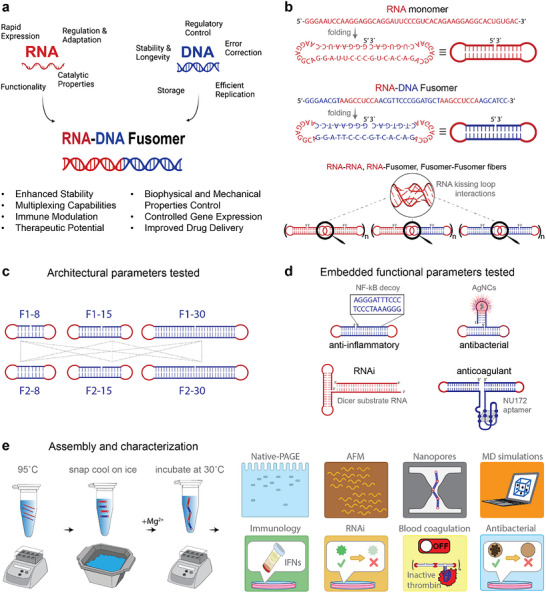
Schematic overview of fusomer design, production, and characterization. (a) The motivation and rationale for RNA/DNA fusomer development. (b) Fusomer fibers design concept. (c) Architectural variations tested with conserved RNA kissing loops and varying DNA stem regions. (d) Examples of functional parameters incorporated. (e) Experimental and computational workflow used in this study for assembly and characterization of fusomer fibers.

In this proof‐of‐concept study, we introduce a new generation of RNA‐DNA fusomer fibers, composed of short chemically synthesized sequences that integrate the functionalities of RNA and DNA within a single nanostructure (Figure 1a). The fiber connectivity was chosen based on previous findings emphasizing the low immunorecognition of these architectures. This design leverages RNA's ability to self‐assemble via HIV‐1‐like kissing loops (KLs) and DNA's intrinsic structural stability and low production cost, while uniting the diverse biological functionalities of both biopolymers (Figure 1b,c) [34, 35, 36, 37]. We demonstrate the tunability of fusomer fibers’ physicochemical, mechanical, and immunological properties, evaluate their cellular uptake in 3D organ‐on‐a‐chip models, and perform molecular dynamics simulations for several representative structures (Figure 1e). We show that simple functionalization of fusomer fibers enables the delivery of multiple therapeutic moieties of both RNA and DNA origin, providing customizable properties and cost‐effective synthesis. Furthermore, we highlight several biomedically relevant applications of fusomer fibers, including regulated gene silencing, intracellular protein inactivation, blood anticoagulation, antibacterial efficacy, and biosensing (Figure 1d).

## 2 Materials and Methods

Detailed procedures for all the experiments are described in the Supporting Information.

### 2.1 Design and Nomenclature of RNA‐DNA Fusomer Fibers

Individual monomers of fusomer fibers were designed to fold into dumbbell‐like structures featuring 9‐nts RNA KLs on either side of a dsDNA stem, which can vary in length and incorporate specific functionalities. Correct folding and exposure of the RNA KLs enables magnesium‐dependent assembly into fibrous nanostructures. Three stem lengths were tested: 8, 15, and 30 base pairs (bps). Fusomer fibers were also functionalized with embedded nuclear factor kappa‐B (NF‐κB) decoy sequences, and appended Dicer‐substrate (DS) RNAs targeting different genes, antibacterial DNA‐templated and stabilized silver nanoclusters (AgNCs), and anticoagulant NU172 aptamers. Typically, fibers are composed of two monomers per repeating unit, allowing each monomer to carry distinct functionalities. The nomenclature used in this manuscript specifies the monomer type (RNA = R; fusomer = F) and, for fusomers, the length of the dsDNA stem. For example, F1‐8 refers to monomer 1 as an RNA‐DNA fusomer with an 8‐base‐pair dsDNA stem. For simplicity, fibers composed of RNA‐DNA fusomer monomers are referred to as fusomer fibers, whereas those composed of RNA monomers, used as controls, are referred to as RNA fibers.

### 2.2 Production and Storage of Fusomer Fibers

For assembly of fusomer fibers and RNA fibers, oligonucleotide strands were combined in equimolar ratios with HyPure cell culture‐grade water and assembled in a one‐pot thermal anneal. Fusomer fibers and RNA fibers were stored at 4°C until use. To confirm successful assembly, 8% native‐PAGE (37.5:1 acrylamide:bis‐acrylamide) was used for visualization.

### 2.3 Dehydration and Stability

For long‐term storage and shipping purposes, the fusomer fibers and RNA fibers stability was assessed upon dehydration and rehydration following an established protocol [29] with minor modifications. Two dehydration methods were tested: lyophilization and vacuum concentration. By the end of the experiment all rehydrated samples and all control samples were visualized on a native‐PAGE or stored at 4°C until needed.

### 2.4 Serum Stability Assay

To assess the relative stability of the materials under biologically relevant conditions, susceptibility to nuclease degradation was evaluated. Fusomer and RNA fibers (at 0.5 µm each) were incubated in 10% fetal bovine serum at 37°C. Aliquots were collected at designated time points, mixed with native‐PAGE loading buffer, and snap‐frozen on dry ice. Samples were subsequently analyzed as described above.

### 2.5 AFM Imaging of Assembled Fusomer Fibers

Samples were diluted at 4°C with assembly buffer and immediately deposited onto amino‐propyl‐silatrane (APS) modified mica [38, 39]. Imaging was performed with a MultiMode AFM Nanoscope system (Bruker Instruments, Santa Barbara, CA, USA) in Tapping Mode at ambient conditions [40, 41]. Images were processed and analysis of the curvature of the resultant nanostructures was done using a “Kappa” plugin with Fiji software [42]. Statistical histograms of the data points were plotted with MagicPlot 3.0 Pro software and further fitted by Gaussian functions.

### 2.6 Molecular Dynamics (MD) Simulations

#### 2.6.1 Coarse‐Grained Simulations of RNA Polymers

Coarse‐grained RNA‐RNA molecular dynamics (MD) simulations were performed using the sequence‐dependent oxRNA2 model [43, 44]. Monomeric RNA strands R1 and R2 (R1‐15 bp and R2‐15 bp have different sequences) were constructed using Oxview [45]. Each strand was minimized for 20 000 steps followed by a subsequent relaxation for 3.06 ns, employing an Andersen‐like John thermostat at 310 K (37°C) and 1 m monovalent salt using a 15‐fs integration timestep.

#### 2.6.2 All‐Atom MD Simulations

All‐atom MD simulations were performed using NAMD2.14 [46] using 2 fs integration timestep and 2‐2‐6 multiple time stepping. Bsc1, OL3, and ffSB14 parameters were used for DNA [47], RNA [48], and proteins [49], respectively, TIP3P for water [50], with CUFIX corrections to model interactions between ions and nucleic acid/proteins [51].

#### 2.6.3 A/B‐Form Analysis

Structural propensity of the fusomer fibers toward an A‐form or B‐form geometry was evaluated by calculating the average rise per base pair in defined regions of the fusomer fibers. Reference values of 0.26 and 0.34 nm were used for canonical A‐form RNA and B‐form DNA, respectively. Each fusomer fiber was partitioned into four subdomains: two segments between the KLs and the nicked junction, and two segments spanning from the nick to the KLs on the opposite strand. For each subdomain, rise per base pair was computed by determining the center‐of‐mass (COM) distance between consecutive base pair helical centers and averaging over the length of the segment.

### 2.7 Immunorecognition of Fusomer Fibers

#### 2.7.1 Primary Human Peripheral Blood Mononuclear Cells (PBMCs) for Cytokine Analysis

PBMCs were isolated from the whole blood of three healthy donor volunteers, which were collected into Li‐herapin‐containing vacutainers under the IRB‐approved NCI‐Frederick protocol OH99CN046 D, NCT # NCT00339911. This protocol was designed to establish and operate a Research Donor Program that meets the requirements for the protection of human subjects from research risks, as detailed in the NIH Multiple Projects Assurance with the OPRR, and is in compliance with the Code of Federal Regulations, Public Welfare, 45 CFR 46: Protection of Human Subjects. Office for Protection from Research Risks, U.S. Government Printing Office, Washington, DC, 1997. Informed consents were obtained from all human participants of the NCI‐Frederick Research Donor Program. To assess the immunorecognition of fusomer fibers by PBMCs, previously established protocols were followed [52]. All fusomer fibers were compared to a representative panel of NANPs (RNA cubes, DNA cubes, and RNA fibers) from prior studies [25]. NANPs and fusomer fibers were tested at a final concentration of 10 nm and were complexed with Lipofectamine 2000 (L2K) at room temperature for 30 min before transfection. Each condition was tested in duplicates for each donor. Positive controls included lipopolysaccharide (LPS) (20 ng/mL), ODN2216 (5 µg/mL), and PHA‐M (10 µg/mL), while 1× PBS served as a negative control. Supernatants were analyzed using a 15‐plex cytokine assay according to the manufacturer's instructions. Assay readouts were obtained using a Quansys ImagePro reader with Q‐view software, and cytokine elevations of ≥2‐fold above baseline were considered physiologically relevant.

#### 2.7.2 Reporter Cell Lines

The following immune reporter cell lines were used to investigate the mechanistic immunorecognition of fusomer fibers and RNA fibers: THP‐1 Dual, HEK‐Lucia RIG‐I, HEK‐Blue hTLR3, HEK‐Blue hTLR7, and HEK‐Blue hTLR9. Cells were plated and transfected with their respective positive controls, fusomer fibers, and RNA fibers at a final concentration of 10 nm complexed with L2K. Cells were incubated for ∼24 h, after which immune reporter activity and cell viability were assessed.

#### 2.7.3 cGAS‐STING Pathway Mechanistic Studies

To confirm fusomer fiber‐specific cGAS‐mediated activation of interferon regulatory factors (IRF) in THP‐1 Dual cells, the small‐molecule inhibitor G140 was used at 10 nm. Cells were pretreated with G140 for 3 h, followed by treatment with F1‐30:F2‐30 fusomer fibers and incubation for an additional 24 h. IRF activation was quantified using the QUANTI‐Luc 4 Lucia/Gaussia assay, and cell viability was measured in parallel using an MTS assay.

#### 2.7.4 Cellular Uptake Mechanistic Study

To confirm the cellular uptake mechanism of fusomer fibers complexed with L2K, MDA‐MB‐231 cells were transfected with fusomer fibers labeled with Alexa Fluor 488 at a final concentration of 100 nm, complexed with L2K. Two temperature conditions were tested, where one plate was placed at 4°C and the other at 37°C. Cells were incubated for 24 h post‐transfection before flow cytometry analysis.

### 2.8 Assessment of Fusomer Fibers’ Uptake in 3D Organ‐on‐a‐Chip

#### 2.8.1 OrganoPlate Culture

OrganoReady Colon Caco‐2 plates were prepared and used to assess uptake of fluorescently labeled fibers. The plate contained 64 chips made of Caco‐2 tubules seeded against collagen I [53, 54, 55, 56]. The tubules formed a leak‐tight barrier that is used to assess the integrity of the barrier of the 3D culture [57]. IRD 800 labeled RNA fibers and fusomer fibers were assembled, characterized, and complexed with DOTAP/DOPE or L2K for 30 min at room temperature (RT, 25°C), before adding media to reach the final volume. The final concentrations tested were 20, 50, and 100 nm. Controls included carriers alone and untreated cells.

#### 2.8.2 Transepithelial Electrical Resistance (TEER) Analysis

To measure the barrier integrity of the Caco‐2 tubules, TEER values were measured. TEER values provided sensitive measurements of the tightness of the tubules, and insights about the toxicity of the transfected fusomer fibers and RNA fibers by assessing their barrier‐induced disruption at the end of the experiment.

#### 2.8.3 Imaging

Twenty‐four hours after transfection, cells were prepared and stained with NucBlue fixed cell ReadyProbes reagent for imaging. OrganoPlate Cytation 5 multi‐mode microplate reader was used for imaging, DAPI and CY5 filters were used at 4X. Fiji software was used for image analysis and uptake quantification.

#### 2.8.4 Nanopore Sensing of Fusomer Fibers and Fusomer‐Protein Complexes

High‐stress silicon nitride (250 MPa SiN) membranes supported by a Si chip as substrates for nanopore fabrication were used. A JEOL 2010F transmission electron microscope was used as previously described [58]. After cleaning, nanopore chips were assembled in a custom flow cell equipped with Ag/AgCl electrodes. Samples were added to the cis (grounded) electrode and positive or negative voltage was applied to the trans chamber. Ionic current through the nanopore was measured using either an Axopatch 200B amplifier digitized at 250 kHz sample rates or a Chimera VC100 amplifier (Chimera Instruments LLC) [59] digitized at 4.17 MHz sample rates. Data analysis was carried out using Pyth‐Ion software (https://github.com/rhenley/Pyth‐Ion/) for loading, low‐pass filtering, and extracting event parameters. Igor Pro (Wavemetrics) was employed in plotting.

### 2.9 Inactivation of NF‐κB by Fusomer Fibers

#### 2.9.1 Primary Human PBMCs for Cytokine Secretion Analysis

In similar experiments to those described above, NF‐κB down regulation was assessed following spiking of plates with LPS. The supernatants for the positive controls were pooled in a 1:1:1 ratio prior to use in the multiplex plates. 24 h after transfecting the cells with the NANPs, 20 ng of LPS was added to all treatments. After spiking the plate with LPS, plates were incubated for 20 hs at 37°C and 5% CO_2_. Afterward, the plate was spun at 400 X G for 5 min, and the supernatants were transferred and analyzed using a 15‐multiplex plate following the manufacturer's protocol.

#### 2.9.2 Immunofluorescence Analysis for NF‐κB Activation

RAW 264.7 macrophage‐like cells were cultured on slides and reverse‐transfected with fusomer fibers using Lipofectamine RNAiMAX transfection reagent and incubated with the fusomer fiber–RNAiMAX complexes for 6 to 24 h prior to treatment with LPS (100 ng/mL) for an additional 6 to 24 h. After treatment, cells were stained and imaged using an EVOS fluorescence microscope.

#### 2.9.3 Quantitative Reverse‐Transcription Polymerase Chain Reaction (qRT‐PCR) Analysis

Total RNA was extracted using TRIzol Reagent according to the manufacturer's protocol. Total RNA was reverse transcribed to complementary DNA (cDNA) using a high‐capacity RNA‐to‐cDNA kit. The expression levels of mRNA encoding COX2 and iNOS were quantified using SYBR green fluorescence and the StepOnePlus real‐time PCR system, using the default cycling conditions recommended by the manufacturer. Each sample was run in triplicate and *ActB* and *Hprt* gene expression was used as endogenous controls. Fold change was calculated using the comparative CT method (2−ΔΔCT). The primer sequences for gene products of interest are described in Table S1.

#### 2.9.4 Antibacterial Activity of Fusomer Fibers

*Staphylococcus aureus* (*S. aureus*) UAMS‐1 was grown from single colonies in LB at 37°C overnight. Bacteria were diluted to 100K bacterium per well in a sterile 96‐well plate with a final volume of 100 µL. Samples were added to the bacteria with a concentration gradient. Initial optical density at 600 nm measurements were taken before incubation at 37°C for 20 h. After incubation, the optical density at 600 nm was taken again to evaluate the minimum inhibitory concentration (MIC). Cells alone, antibiotic, and concentrations at and above the MIC were plated for CFU (Colony‐Forming Unit). The following day, colonies were counted on each plate to find the minimum bactericidal concentration (MBC) with at least 99.9% bacteria cell death [21]. For excitation‐emission matrix (EEM) analysis, fusomer fibers with AgNCs and C12 AgNCs were synthesized at 10 µm. After overnight incubation, 100 µL of each sample was transferred to a 96‐well black‐walled plate. Emission spectra were recorded from 350 to 700 nm after excitation across wavelengths of 400–850 nm.

#### 2.9.5 Anticoagulant Activity of Fusomer Fibers

Coagulation parameters, including prothrombin time (PT), activated partial thromboplastin time (APTT), and thrombin time (TT), were measured using clinical‐grade reagents and instrumentation. Peripheral blood was collected and processed from ten healthy adult donors under the FMUSP ethical protocol NP 1378/18. For each condition, fusomer fibers with NU 172 aptamer (NU fusomer fiber; final concentration of 3 µm) were added to pooled human plasma in 1.5 mL microcentrifuge tubes. Samples were incubated at 37°C for 30 min. Coagulation was initiated by automated addition of the respective reagents on an ACL TOP 350 CTS analyzer, and clotting times were recorded in seconds. While statistical differences in clotting time were observed between treated and control samples, the short duration and high precision of these assays (CV <5%) limit their interpretability as a direct measure of biological effect.

#### 2.9.6 Specific Gene Silencing by Fusomer Fibers

Prior to transfection, fusomer fibers carrying dicer substrate (DS) RNAs against green fluorescent protein (GFP) [60] were assembled at a 1 µm concentration. These constructs were then complexed with L2K and incubated at room temperature for 30 min. MDA‐MB‐231 eGFP cells were transfected with the complexes at final concentrations of 10 or 50 nm. Following transfection, cells were incubated at 37°C with 5% CO_2_ for 72 h, after which GFP expression was visualized using an EVOS cell imaging system. DS RNA against GFP was used in all experiments as a control.

#### 2.9.7 Statistical Analysis

Experimental data were normalized to controls and are presented as mean ± SEM (or SD, as indicated). The number of biological and technical replicates for each experiment, and the test used for statistical analysis is specified in the corresponding figure legends. Statistical analyses were performed using GraphPad Prism with *P* < 0.05 considered statistically significant.

## 3 Results and Discussion

### 3.1 Fusomer Fibers Have Tunable Structural and Mechanical Properties

**FIGURE 2:**
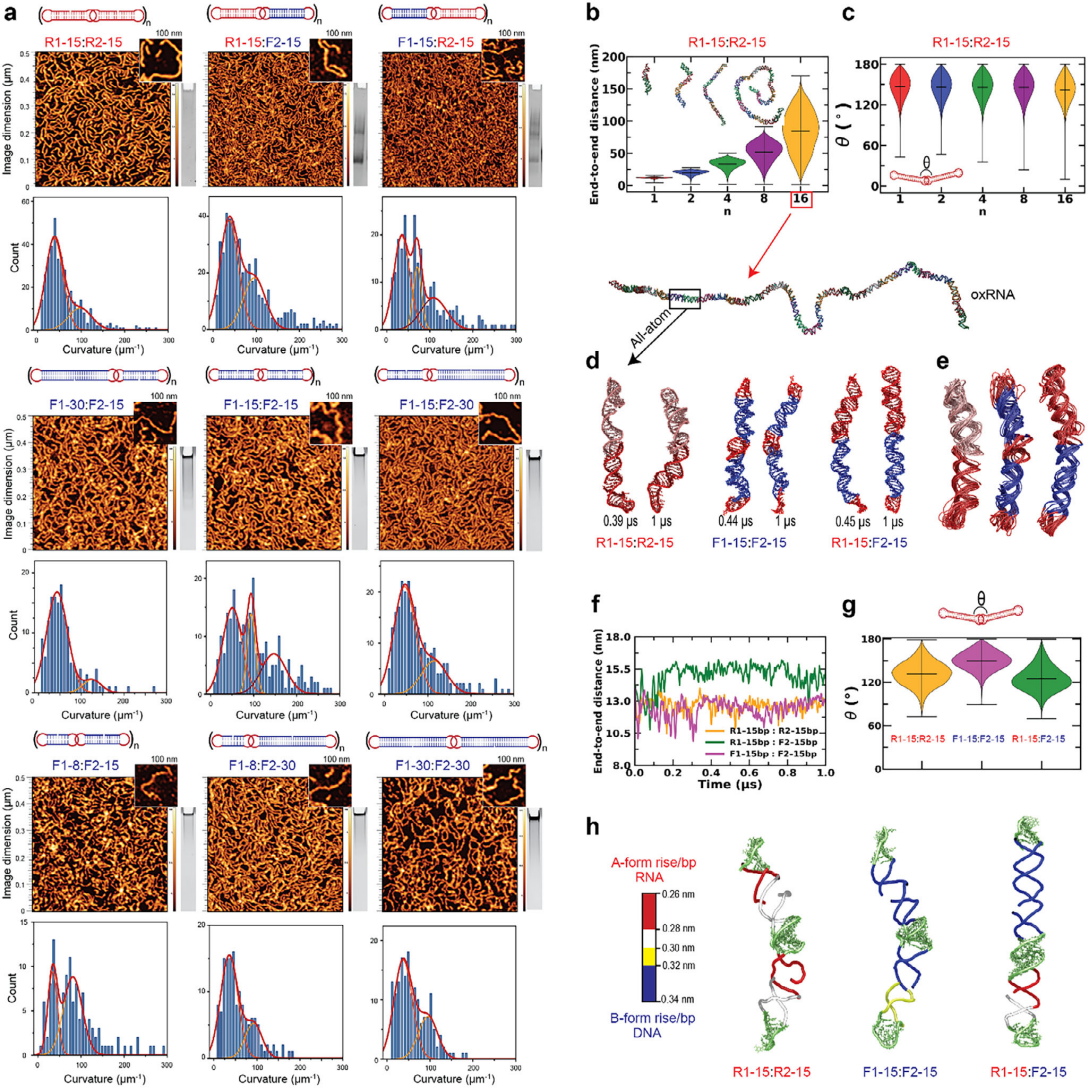
Characterization of the fusomer fibers. (a) Representative AFM topography images of RNA and fusomer fibers with varying stem lengths showing mechanical properties tunability. (b) End‐to‐end distance and (c) angle between the arms at the KLs junction in oxRNA simulations of different length RNA fibers. (d) Representative instantaneous conformations and (e) overlayed snapshots of backbone traces in all‐in atom MD simulations of the fibers. Snapshots were selected every 50 ns. (f) End‐to‐end distance versus simulation time. (g) Distribution of arms’ angle at the KLs junction in R1‐15:R2‐15, F1‐15:F2‐15, and R1‐15:F2‐15 averaged over 1µs all‐atom simulation. (h) Local propensity for A or B form helix classified according to the average rise/bp value. RNA KLs are shown in green.

All fusomer monomers were pre‐folded to self‐assemble into fibrous structures via RNA KLs. DNA stems of varying lengths were tested to evaluate the assembly and tunability of fibers’ mechanical properties. Successful assembly was confirmed by native‐PAGE, while morphological and mechanical characterization was performed by AFM (Figure 2a) [61]. Topological statistical analysis of structural conformations, in many cases, shows a perfect agreement with the mechanical properties of nanostructured objects [62, 63, 64, 65]. AFM imaging of tested fusomer fibers revealed fibrous nanostructures of a continuous nature that also appeared curved: a central morphological feature of many biological structures [66]. For materials with predominantly linear morphology, the curved structure could indicate the degree of mechanical robustness, as well as internal structural organization [67, 68]. It was noticed that the appearance of the nanostructures varied with the composition of the monomeric building blocks. To quantify the mechanical properties of different fusomer fibers, the degree of curvature (*κ*) was measured at multiple points along each individual structure. The curvature at a given point, defined as the inverse of the radius of the “oscillating circle”, fitted to the arc of the curve [69], represents the local bending rigidity of a fusomer fiber. Larger *κ* values correspond to regions of greater curvature and reduced stiffness. The *κ* values were collected, averaged, and plotted as statistical histograms, revealing a broad distribution of curvature that reflects the heterogeneity of the nanostructures and highlights how monomer composition influences both structural order and macroscopic mechanical properties.

There are two major contributing factors to fusomer fibers and RNA fibers flexibility: i) their composition, distinguishing between RNA and DNA helices, and ii) the density of the KLs along the polymer chain, defined by the length of stems. The impact of polymer composition suggests that substituting both RNA monomers with fusomers (*e.g*., F1‐15:F2‐15) significantly enhances flexibility, as indicated by the broader distribution of *κ*‐values in the histograms. The average *κ* value for the R1‐15:R2‐15 fibers was 62 µm^−1^, significantly lower than the 125 µm^−1^ observed for the F1‐15:F2‐15 fibers. Introducing just one fusomer monomer (F15) into the chain, either as the first (F1‐15:R2‐15, *κ* = 81 µm^−1^), or second (R1‐15:F2‐15, *κ* = 92 µm^−1^) monomer, resulted in intermediate curvature values, as expected. Further analysis involved fitting Gaussian functions to the curvature value distribution, providing insights into the relative prevalence of straight versus curved regions of the fibers. The first Gaussian peak corresponded to straighter nanostructures, while subsequent peaks at higher *κ*‐values represent contributions of more curved, flexible chains. The presence of flexible nanostructures became more pronounced with the introduction of fusomer fibers, as indicated by the increased *κ* values.

Next, the influence of KL density on the fusomer fibers’ flexibility was explored. Given that KLs act as hinges, higher densities per unit length were anticipated to reduce fiber stiffness. Starting with a composition of F1‐15:F2‐15 (*κ* value = 125 µm^−1^), monomer one was replaced with F1‐30 (F1‐30:F2‐15), lowering the density of KLs in each structure. This replacement produced noticeably stiffer nanostructures (F1‐30:F2‐15, κ‐value = 58.0 µm^−1^). Further exchange of monomer one with F1‐8, increased the density of KLs and resulted in more flexible fibers (F1‐8:F2‐15, *κ* = 86.7 µm^−1^). The stiffness increased again when the second monomer F2‐15 was replaced with F2‐30, reducing the KL density (F1‐8:F2‐30, *κ* = 57 µm^−1^). Interestingly, further swapping F1‐8 for F1‐30 (F1‐30:F2‐30) resulted in a somewhat higher *κ* value (*κ* = 83.0 µm^−1^) than expected. This behavior is likely due to two competing effects: the reduced KL density, which should stiffen the structure, and the longer fusomer fiber segments between KLs, which enhance flexibility. Thus, despite F1‐30:F2‐30 having the lowest KL density, the extended fusomer fiber length may account for the observed increase in *κ* value. Overall, the trend was consistent with our expectations, showing that replacing longer monomers with shorter ones enhances KL density and fibers’ flexibility as evidenced by larger curvature values in the statistical distribution.

### 3.2 MD Simulations Confirm Fusomer Fibers Are More Flexible Than RNA Fibers

Coarse‐grained simulations were performed using the oxRNA model following constructs with increased numbers of monomers per fiber (*n* = 1, 2, 4, 8, 16) to further understand the dynamics of RNA fibers. The simulations were found to agree with our experimental observation that longer fibers tend to adopt bent conformations resulting in significantly reduced end‐to‐end distances (Figure 2b) relative to their maximum lengths (contour lengths). For shorter constructs (*n* = 1, 2), the end‐to‐end distance closely matched the expected linear extension, consistent with a relatively rigid and extended duplex geometry. However, beyond *n* = 4, the constructs began to exhibit curvature and looping as seen in the AFM images. At the same time, the inter‐arm angles measured at the KL junctions of RNA fiber constructs remained around ∼150° regardless of the fiber length (Figure 2c). The persistence length (l_p_) of the RNA fiber (calculated from *n* = 16) was ∼23 nm. l_p_ ∼ 2 K_h,_ where K_h_ is the Kuhn's length of the polymer calculated as the mean squared end‐to‐end distance divided by the contour length.

The structural behavior of fusomer fibers was also evaluated using all‐atom MD simulations (Figure 2d,e) for three representative constructs: R1‐15:R2‐15, F1‐15:F2‐15, and R1‐15:F2‐15. Among the three, the hybrid construct R1‐15:F2‐15 had a slightly longer average end‐to‐end distance of ∼15 nm, compared to ∼13 nm for the R1‐15:R2‐15 and F1‐15:F2‐15 constructs (Figure 2f). Interestingly, while the R1‐15:R2‐15 RNA fiber, with a helical rise of ∼0.26 nm per bp, was expected to yield a more compact structure, the F1‐15:F2‐15 fusomer fiber with a helical rise of ∼0.34 nm also exhibited a shorter end‐to‐end distance. This finding is likely due to the greater flexibility of dsDNA compared to dsRNA. The higher flexibility of DNA allows for more pronounced bending resulting in a more compact geometry despite its longer contour length. The angle between the arms at the KLs site averaged around 135° for R1‐15:R2‐15 and R1‐15:F2‐15 constructs and 150° for the F1‐15:F2‐15 construct. This is consistent with oxRNA simulations, where the angle between the arms was ∼150°. The R1‐15:R2‐15 RNA fiber predominantly adopts an A‐form helix, with some segments exhibiting a rise per base pair exceeding 0.28 nm (Figure 2h). A similar effect was observed in the F1‐15:F2‐15 fusomer fiber, although this was less pronounced, as the majority of the DNA duplex maintains a consistent B‐form rise throughout the structure. Similarly, for the R1‐15:F2‐15 fusomer fiber, RNA deviates the most from its ideal geometry.

### 3.3 Dehydrated Fusomer Fibers Can be Stored and Handled at Ambient and Higher Temperatures

Preservation of structural integrity and biological function during storage and shipping remains a critical barrier to the broader clinical application of TNAs. Our team previously developed protocols suitable for long‐term storage of dehydrated NANPs at higher temperatures [29]. Applying this approach to fusomer fibers, several representative structures were dehydrated by either vacuum concentration or lyophilization, stored at 50°C for one week, rehydrated, and analyzed by native‐PAGE (Figure S1). Consistent with previous findings, the dehydration methods preserved fusomer fibers and RNA fibers under heat stress, whereas those stored in solution showed degradation. These protocols are applied for prolonged storage periods and ambient shipping of fusomer fibers, increasing their viability for intended applications.

### 3.4 Relative Chemical Stability of Fusomer Fibers Exceeds RNA Fibers

To evaluate the potential of carrier‐free fusomer fibers for in vivo applications, their stability under biologically relevant conditions was assessed and compared to RNA fibers. As shown in Figure S2, RNA fibers were sensitive to nucleases, with degradation becoming apparent after 1 min. In contrast, incorporation of a DNA stem in fusomer fiber monomers enhanced stability, preserving some structural integrity throughout the 40 min of incubation.

### 3.5 Structure and Composition Define Immunorecognition of Fusomer Fibers

Understanding the structural parameters that influence fusomer fibers’ immunorecognition is essential to ensure their safety in biomedical applications. Furthermore, comparison with other well‐characterized NANPs provides critical context for evaluating fusomer fibers’ immunological profiles and guiding their optimization for potential therapeutic use. To address this, representative fusomer fibers were compared with 3D RNA and DNA NANPs (RNA and DNA cubes, respectively) as well as RNA fibers to evaluate their immunostimulatory responses following transfection into human PBMCs.

**FIGURE 3:**
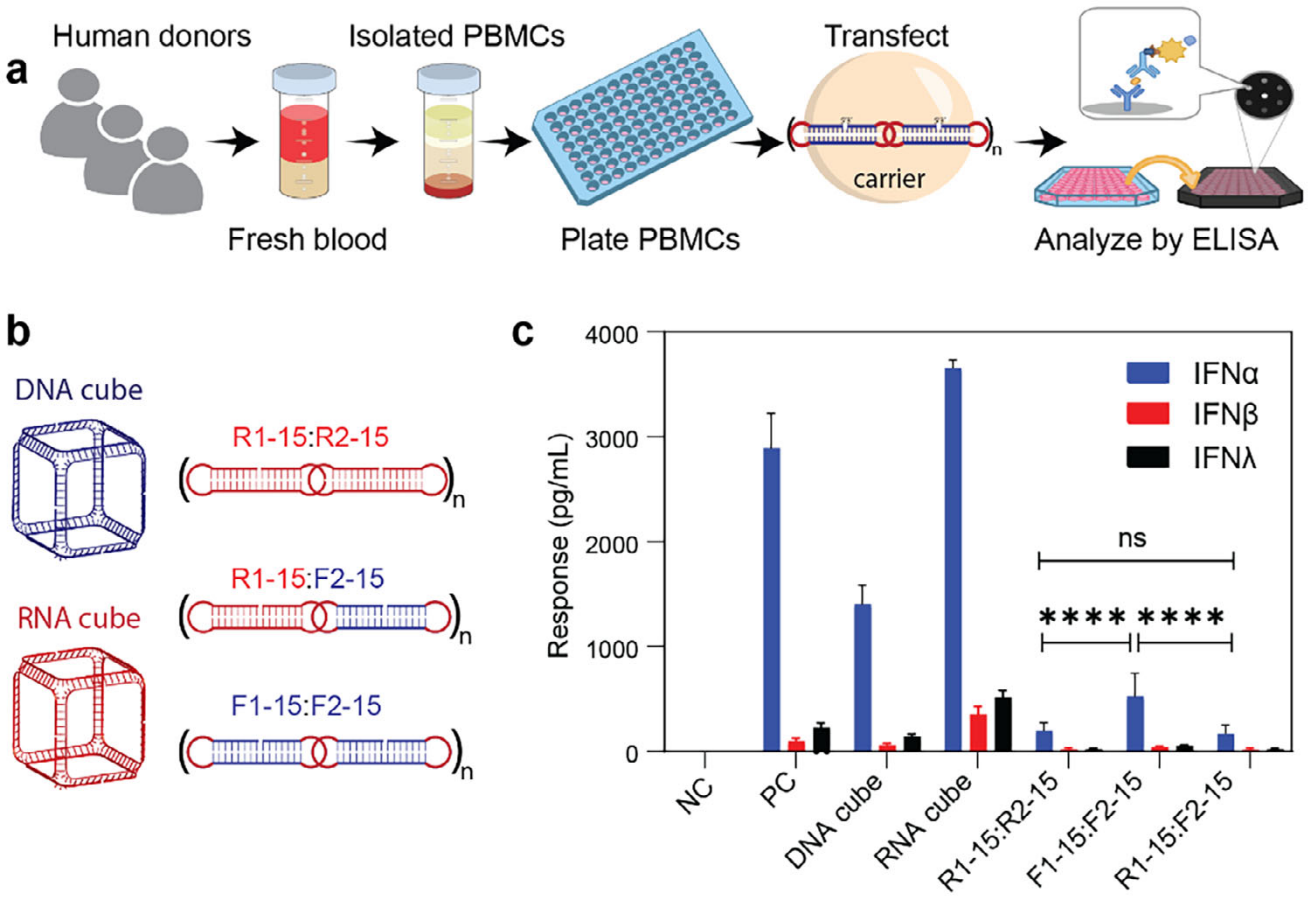
Relative immunorecognition of fusomer fibers. (a) Schematic outline of experimental pipeline. (b) Panel of control NANPs and fusomer fibers tested. (c) Type I and Type III IFN production levels of three healthy donors after treatment with DNA cubes, RNA cubes, RNA fibers (R1‐15:R2‐15), RNA‐fusomer fibers (R1‐15:F2‐15), and fusomer fibers (F1‐15:F2‐15) with positive control ODN 2216. Data is shown as mean ± SEM, (*n* = 3). Statistical significance according to the 2‐way ANOVA highlighted on the figure is for fusomer fibers as compared to other fibrous structures. The difference of RNA cubes compared to DNA cubes, and RNA cubes compared to fusomer fibers for all IFNs, and of DNA cubes compared to fusomer fibers for IFNa, was also significant, but not included in the graph for simplicity. Full statistical analysis is presented in Table S4.

The experimental settings for this model were specifically optimized [52] as PBMCs produce the most predictive and reliable results for potential cytokine storm toxicity, unlike other models commonly used in preclinical research [70, 71, 72]. PBMCs isolated from freshly collected blood of three healthy human donors were incubated for 24 hs with control NANPs, RNA fibers, or fusomer fibers complexed with L2K. The levels of Type I and Type III interferons, well‐established biomarkers of immune activation [25], were measured by a multiplex ELISA (Figure 3 and Figure S3a). While RNA and DNA cubes induced all tested interferon biomarkers in all tested cultures, the resulting interferon levels were higher in the RNA cube‐treated cultures vs the DNA cube‐treated cultures (Figure 3c), in agreement with our previous findings [25, 73, 74]. Fibrous NANPs, including RNA fibers (R1‐15:R2‐15), RNA‐fusomer fibers (R1‐15:F2‐15), and fusomer fibers (F1‐15:F2‐15), did not induce IFNw (Figure S3a), but resulted in a slight increase above the baseline in IFNa, IFNb, and IFNl levels (Figure 3c). Among these constructs, fusomer fibers were more potent than RNA fibers and RNA‐fusomer fibers, each of which induced comparable but slightly lower levels of IFNa, IFNb, and IFNl (Figure 3c). The difference in IFNa levels of RNA fibers compared to fusomer fibers and RNA‐fusomer fibers compared to fusomer fibers was statistically significant (*p* < 0.0001, Figure 3c). Differences in IFNa levels between RNA fibers and RNA‐fusomer fibers were not statistically significant. The differences in IFNβ and IFNλ levels between all fusomer fibers were not statistically significant (Table S4). These responses were consistent in cultures from three donors.

Additionally, several reporter cell lines were utilized to investigate potential mechanisms of fusomer fiber immunorecognition by pattern‐recognition receptors (PRRs), which detect nucleic acids and trigger innate immune responses. First, fusomer fibers were evaluated against THP‐1 Dual reporter cells, engineered with reporter genes driven by key immune signaling components NF‐κB and IRF. As shown in Figure S3b, minimal NF‐κB activation was observed following treatment with R1‐15:R2‐15 or F1‐15:F2‐15, F1‐30:F2‐30, or F1‐15:F2‐30 fibers, consistent with our previous findings [75]. In contrast, activation of the IRF pathway was induced by F1‐15:F1‐15, F1‐30:F2‐30, and F1‐15:F2‐30 fibers, but not by R1‐15:R2‐15 fibers, most likely due to cytosolic cGAS‐STING pathway activation by dsDNA [22, 75, 76]. Interestingly, F1‐30:F2‐30 fusomer fibers exhibited stronger immunostimulatory activity than F1‐15:F2‐30 fusomer fibers, suggesting a length‐dependent mechanism of cGAS‐STING activation [77, 78]. To confirm this mechanism, a selective inhibitor of cGAS was applied, which significantly reduced fusomer fiber‐induced IRF activation (Figure S4). These results support the conclusion that cGAS serves as the primary sensor driving fusomer fiber recognition and IRF pathway activation in THP‐1 Dual cells. These results, along with the AFM image analysis confirm that the flexibility of fibers is linked to length and composition of the fibers, which is linked to their biological function. To complement these studies, the fusomer fibers were also evaluated against HEK‐Blue hTLR3, HEK‐Blue hTLR7, HEK‐Blue hTLR9, and HEK‐Lucia RIG‐I reporter cells, each engineered to express PRRs specific for nucleic acid recognition. As shown in Figure S3b, no significant activation was observed across these systems, indicating that these PRRs are not involved in sensing RNA fibers or fusomer fibers, consistent with results from PBMC assays and prior reports [75]. Collectively, these data support the notion that fusomer fiber immunorecognition can be tailored by modulating their composition and architectures, underscoring the potential for their rational design for biomedical applications.

### 3.6 Fusomer Fibers Cellular Uptake via Endocytosis

To confirm delivery efficiency and elucidate the cellular uptake mechanism of fusomer fibers complexed with L2K, ATP‐dependent processes, including endocytosis, were evaluated by incubating transfected cells at 4°C and 37°C. Flow cytometry analysis revealed a clear temperature dependence of fluorescently labeled fusomer fiber uptake in MDA‐MB‐231 cells. As shown in Figure S5, cells incubated at 37°C exhibited a shift in fluorescence intensity, indicating efficient uptake, whereas incubation at 4°C showed no appreciable shift relative to background controls, with minimal fluorescent cells detected. Since low temperatures inhibit ATP‐dependent processes, including endocytosis, the absence of uptake at 4°C indicates that internalization is energy dependent. These findings suggest that fusomer fibers are predominantly internalized via endocytic pathways when delivered with L2K, rather than through passive or temperature‐independent mechanisms, consistent with our previously published results [25].

### 3.7 Fusomer Fibers Can be Efficiently Delivered Into 3D Organ‐on‐a‐Chip

**FIGURE 4:**
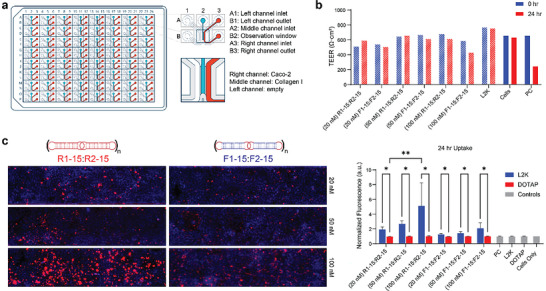
Delivery of RNA fibers and fusomer fibers into 3D organ‐on‐a‐chip. (a) Schematic layout of a 3L64 OrganoReady Colon Caco‐2 plate, displaying the channels in each chip, and demonstrating how the Caco‐2 tubule is seeded against the collagen‐I. Every chip included a right channel inlet and outlet (A3, B3), a left channel inlet and outlet (A1, B1), a gel channel (A2), and an observation window (B2) showing the intersection of the three channels for imaging (b) Transepithelial electrical resistance (TEER) measurements following treatment with RNA fibers and fusomer fibers complexed with L2K. Control conditions include vehicles alone, and positive control that disrupts the barrier integrity. TEER values are presented as mean ± SEM, (*n* = 3). (c) Representative microscopy images of OrganoReady Colon Caco‐2 cultures at 4x magnification, showing fluorescent uptake of RNA fibers and fusomer fibers complexed with L2K after 24 h The images show that the uptake is concentration dependent. The intensity of the IRD800 signal of fusomer fibers was quantified and presented as mean ± SD, (*n* = 3). Statistical significance according to the 2‐way ANOVA highlighted on the figure.

In recent years, 3D cultures have gained appreciation and translational relevance, especially in the context of new approach methodologies (NAMs), as a tool that allows better recapitulation of the structural and biochemical complexities of human tissues compared to traditional 2D cell cultures. To assess the relative uptake of fusomer fibers in 3D culture, a 3‐lane 64 OrganoPlate was seeded with Caco‐2 culture forming tubules. The plates used were standard 384‐well plates, comprising 64 chips, each chip being made of 2 × 3 blocks of wells (Figure 4a). The fusomer and RNA fibers were complexed with L2K or DOTAP/DOPE and transfected into the Caco‐2 tubules to measure their relative uptake and assess their effect on barrier integrity at 24 h.

TEER measurements were used to indicate the toxicity of the treatments to the Caco‐2 tubules, which was achieved by assessing the induced disruption of the tubule barrier over time. Lower TEER values indicate barrier integrity disruption induced by the treatments. As shown in Figure 4b, the TEER measurements indicated that the Caco‐2 tubule barrier integrity was not disrupted by vehicle controls or any of the treatments, compared to the positive control after 24 h of transfection. This supports the notion that fusomer and RNA fibers are not cytotoxic. In addition, fluorescence imaging of the labeled fusomer and RNA fibers showed that the uptake was concentration dependent (Figure 4c). Interestingly, the fusomer and RNA fibers complexed with L2K showed better cellular uptake than fusomer and RNA fibers complexed with DOTAP/DOPE, with this difference particularly apparent at the lowest transfection concentration employed (20 nm). Furthermore, the relative uptake of RNA fibers was found to be significantly higher than that of fusomer fibers when complexed with L2K.

### 3.8 Anti‐Inflammatory Fusomer Fibers Attenuate NF‐κB Activity in Human Cells

**FIGURE 5:**
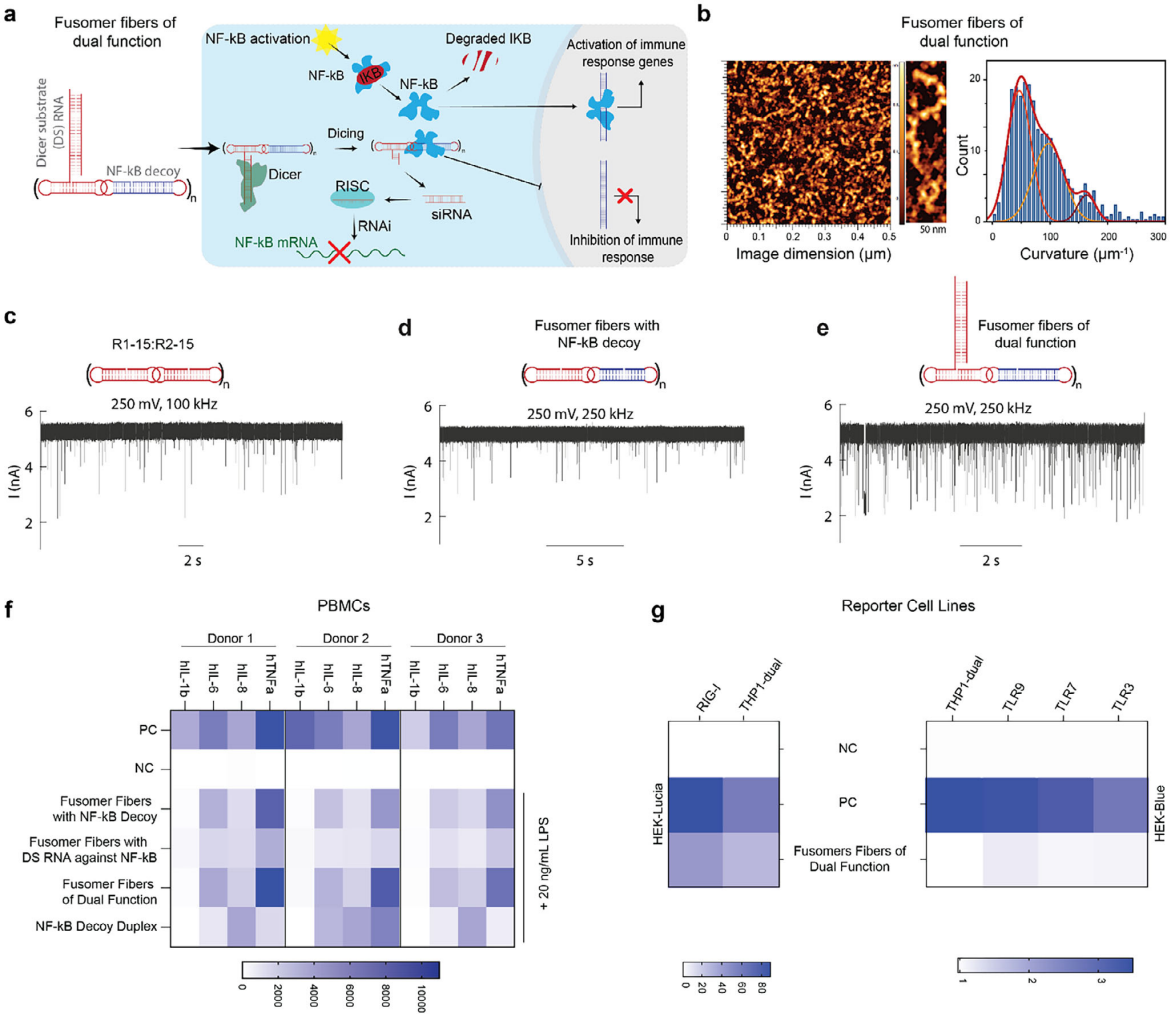
Assessment of fusomer fibers with dual function. (a) Schematic outlines the design of dual function fusomer fibers, and their activity in the cell for NF‐κB downregulation. (b) AFM topography image of dual function fusomer fibers. (c–e) Characterization of different fibers with nanopores, current traces for (c) RNA fiber were recorded at a sampling rate of 250 kHz and low pass filtered at 100 kHz, but current traces for (d,e) hybrid fibers were recorded at a sampling rate of 4167 kHz and low pass filtered at 250 kHz. Concentration of RNA fiber was 65 nm. Concentration of fusomer fibers with NF‐κB decoy and dual function fusomer fibers was 71 nm each. (f) Cytokine production upon PBMCs treatment with dual function fusomer fibers, fusomer fiber with NF‐ĸB decoy, and fusomer fibers with DS RNA against NF‐ĸB, and control NF‐κB decoy duplex. (g) Immune recognition of dual function fusomer fibers assessed in reporter HEK‐Blue hTLR3, HEK‐Blue hTLR7, HEK‐Blue hTLR9, HEK‐Lucia RIG‐I, and THP1‐Dual cell lines. Data is presented as mean (*n* = 3).

The NF‐κB pathway plays pivotal physiological and pathological roles ranging from inflammatory responses, cell survival, and stress responses, to chronic inflammation and cancer progression [79]. NF‐κB is a transcriptional factor that regulates proinflammatory cytokines, chemokines, enzymes, adhesion molecules, and receptors. It is inactive when bound to inhibitory proteins (IκB) and sequestered in the cytoplasm [80]. Upon exposure to stimuli, IκB is phosphorylated and degraded, allowing for the nuclear translocation of the activated NF‐κB subunits, where they bind to the κB consensus sequence in the promoter regions of proinflammatory genes to initiate their transcription [23, 79]. Hence, dual‐function fusomer fibers were designed to downregulate inflammatory responses by (i) delivering embedded dsDNA decoys, that mimic the κB consensus sequence, which bind activated cytosolic NF‐κB and prevent its translocation to the nucleus [23, 81], and (ii) delivering Dicer substrate (DS) RNAs, which undergo intracellular Dicer‐assisted release of therapeutic siRNAs targeting translation of NF‐κB [30, 82]. Consequently, these dual‐function fusomer fibers can simultaneously inhibit activated NF‐κB through decoys and suppress NF‐κB expression via RNAi, thus achieving the therapeutic goal of limiting pro‐inflammatory activity. Figure 5a illustrates the design of dual‐function fusomer fibers, in which one monomer incorporates the NF‐κB decoy and a second monomer carries DS RNA targeting NF‐κB. The figure also depicts the mechanism of NF‐κB downregulation in cells. The synthesized dual function fusomer fibers were characterized by AFM imaging. In addition, SiN_x_ nanopores were used to characterize and compare the RNA fiber (unbranched structure, Figure 5c), fusomer fiber with NF‐κB decoy (unbranched structure, Figure 5d), and fusomer fibers of dual function (branched structure, Figure 5e). Two different nanopores with similar sizes, based on the pore conductance, were used: one to characterize the RNA fibers and the other to characterize unbranched and branched fusomer fibers. RNA fibers produced fast events with broad distribution in current blockades while passing through ∼5.5 nm (Figure 5c and Figure S6a), as shown in a previous study [83]. Interevent time distributions were fit to exponential decay functions to extract the mean capture rates and these increased with voltage (Figure S6b). A similar behavior was observed in the unbranched fusomer fibers (Figure 5d and Figure S6c,d). The branched fusomer fibers produced two distinct populations, one like the unbranched fusomer fiber and another with deeper and longer events (Figure 5e and Figure S6e,f). This observation is consistent with findings in a previous study of unbranched and branched RNA fibers [83]. Figure S6e also shows the shift of event population toward the lower dwell time region when the voltage was increased from 250 to 300 mV, which suggests that the fibers translocate through the nanopore. The capture rate for branched fusomer fibers (Figure S6f) was greater than that for unbranched analogs at the same concentration, suggesting that the branched fusomer fibers were easily detectable using our high‐bandwidth electronics.

Next, dual‐function fusomer fibers containing NF‐κB decoys and DS RNAs targeting NF‐κB were evaluated for their ability to modulate NF‐κB activity in human PBMCs. Cytokine responses were measured using a multiplex panel that included type I and III interferons regulated by IRFs, as well as key NF‐κB‐dependent inflammatory cytokines, including TNF‐α, IL‐1β, IL‐6, and IL‐8. A combination of LPS, ODN 2216, and phytohemagglutinin (PHA‐M) was used as a positive control. Fusomer fibers of dual function, fusomer fibers with NF‐ĸB decoys, fusomer fibers with DS RNAs against NF‐ĸB, and free NF‐κB decoy duplexes were tested for their ability to interfere with PBMC‐mediated pro‐inflammatory immune responses. Figure 5f and Figure S7 show the resulting responses. The positive control induces production of all proinflammatory cytokines as expected based on its known pro‐inflammatory activity [84]. LPS‐induced IL‐1β was downregulated by the dual‐function fusomer fibers, fusomer fiber with NF‐ĸB decoys, fusomer fibers with DS RNAs, and the NF‐κB decoy duplexes in PBMC cultures of all three donors. While IL‐6 and TNF‐α levels were downregulated in all cultures by the four constructs, the NF‐κB decoy duplexes and fusomer fibers with DS RNAs were more potent than dual‐function fusomer fibers and fusomer fibers with NF‐ĸB decoys in cultures of two donors (Donors 1 and 3), whereas the NF‐κB decoy duplexes were less potent in downregulating these cytokines in cultures of one donor (Donor 2). The IL‐8 levels induced by 20 ng/mL LPS were excessively high, preventing detection of any downregulation by the NF‐κB decoy duplex in cultures from the three donors. In contrast, dual‐function fusomer fibers, fusomer fibers containing only the NF‐κB decoys, and fusomer fibers carrying DS RNAs against NF‐κB exhibited a more pronounced reduction of LPS‐induced IL‐8 levels. The goal of this study was to provide proof of concept that functionalizing fusomer fibers, either by embedding NF‐κB decoys, appending DS RNAs, or both, confers the ability to downregulate NF‐κB. More broadly, the study demonstrates that dual functionalization of fusomer fibers preserves their intended biological activity. Overall, these data confirm the functionality of all constructs, and suggest that their complex cell‐entry, release, and both extra‐ and intra‐cellular fate may contribute to the differential response observed in tested PBMC cultures. Genetic polymorphisms in the genes for IL‐1β, IL‐6, IL‐8, and TNF‐α, as well as genes involved in their regulation, are a known cause of inter‐donor variability in the production of these cytokines [85, 86]; therefore, the heterogeneity in the response to fusomer fibers of dual function, fusomer fiber with NF‐ĸB decoy, and fusomer fibers with DS RNA against NF‐ĸB, and control NF‐κB decoy duplex was not unexpected. Follow‐up studies will include additional optimization of incubation times and concentration of treatments, along with release kinetics, to further explore the potential synergistic action of dual function fusomer fibers.

Fusomer fibers of dual function were further characterized for their immune recognition. Reporter cell lines, HEK‐Blue hTLR3, HEK‐Blue hTLR7, HEK‐Blue hTLR9, HEK‐Lucia RIG‐I, and THP1‐Dual, were used to investigate the recognition of constructs by specific PRRs. Figure 5g shows that there was no significant activation of the NF‐κB pathway in the hTLR and THP1‐Dual reporter cells. However, there was activation of the IRF pathway, with greater activation in RIG‐I reporter cells than THP1‐Dual reporter cells, indicating that the addition of the DS RNA moiety in the dual function fusomer fiber may play a role in RIG‐I activation. This observation agrees with recent studies [24, 87].

Nuclear translocation of NF‐κB is a central step in its activation pathway [88, 89]. To evaluate the effect of dual‐function fusomer fibers on NF‐κB localization, we performed immunofluorescence assays in the murine macrophage cell line RAW 264.7, either pretreated or not with our constructs prior to LPS exposure (100 ng/mL for 15 min). Following LPS stimulation, NF‐κB was detected in both the cytoplasm and nucleus, as indicated by green fluorescence distributed throughout the cell. Nuclear regions were identified using Hoechst staining, and nuclear NF‐κB fluorescence intensity was quantified. Notably, cells pretreated with fusomer fibers of dual function exhibited reduced nuclear NF‐κB fluorescence intensity (Figure S8), indicating that fusomer fiber treatment attenuates LPS‐induced NF‐κB nuclear translocation.

*COX2* and *iNOS* genes are downstream NF‐κB regulated genes [90, 91, 92], which play a critical role in inflammation [93], angiogenesis, and tumor survival [94]. RT‐PCR analysis was performed to assess the levels of *Cox2* and *iNOS*‐ gene expression levels upon downregulation of NF‐κB. Reduction in *Cox2* gene expression in RAW 264.7 cells was observed when transfected with fusomer fibers of dual function (fold change = 0.77) prior to LPS stimulation (50 ng/mL for 24 h) (Figure S9a) and a comparable pattern was observed for *iNOS* expression (fold change = 0.75) in cells treated with dual‐function fusomer fibers relative to the control (Figure S9b). These results suggest that fusomer fibers of dual function can attenuate the expression of these key inflammatory mediators induced by LPS stimulation in a higher magnitude than observed with the other constructs. It is important to note, however, that LPS stimulation induced a robust upregulation of *Cox2* and *iNOS* expression, which may have mitigated the inhibition achieved by fusomer fibers treatments.

To confirm sequence specificity and demonstrate that the versatility of fusomer fibers enables facile targeting of different genes, the fibers were functionalized with DS RNAs selected against GFP. Fluorescence microscopy was used to qualitatively assess gene silencing efficacy in breast cancer cells expressing GFP (MDA‐MB‐231/GFP). As shown in Figure S10, treatment with either the functionalized fusomer fibers or the control DS RNAs resulted in a marked reduction of GFP fluorescence compared to untreated cells.

### 3.9 Fusomer Fibers Can be Used for Protein Sensing

Various challenges hinder the sensing of proteins via solid‐state nanopores. This is attributable to the heterogeneous charge distribution of proteins, leading to unpredictable electrophoretic forces and complications in the capture and translocation of proteins through the nanopore [95]. Another challenge is the rapid and variable translocation and dwell times caused by the size of the protein, which is often smaller than the nanopore size, producing a weak signal that is hard to resolve, and decreases the signal‐to‐noise ratio [96, 97, 98]. In addition, the selectivity and accuracy for detecting proteins of same size, or proteins with different post‐translational modifications, is very hard to achieve, as their dwell times or blockade amplitudes can be similar [95]. To overcome these challenges, nucleic acid‐based scaffolds can be used to selectively bind to the target protein and pass through the nanopore as a complex allowing for more reliable and reproducible readouts [99].

**FIGURE 6:**
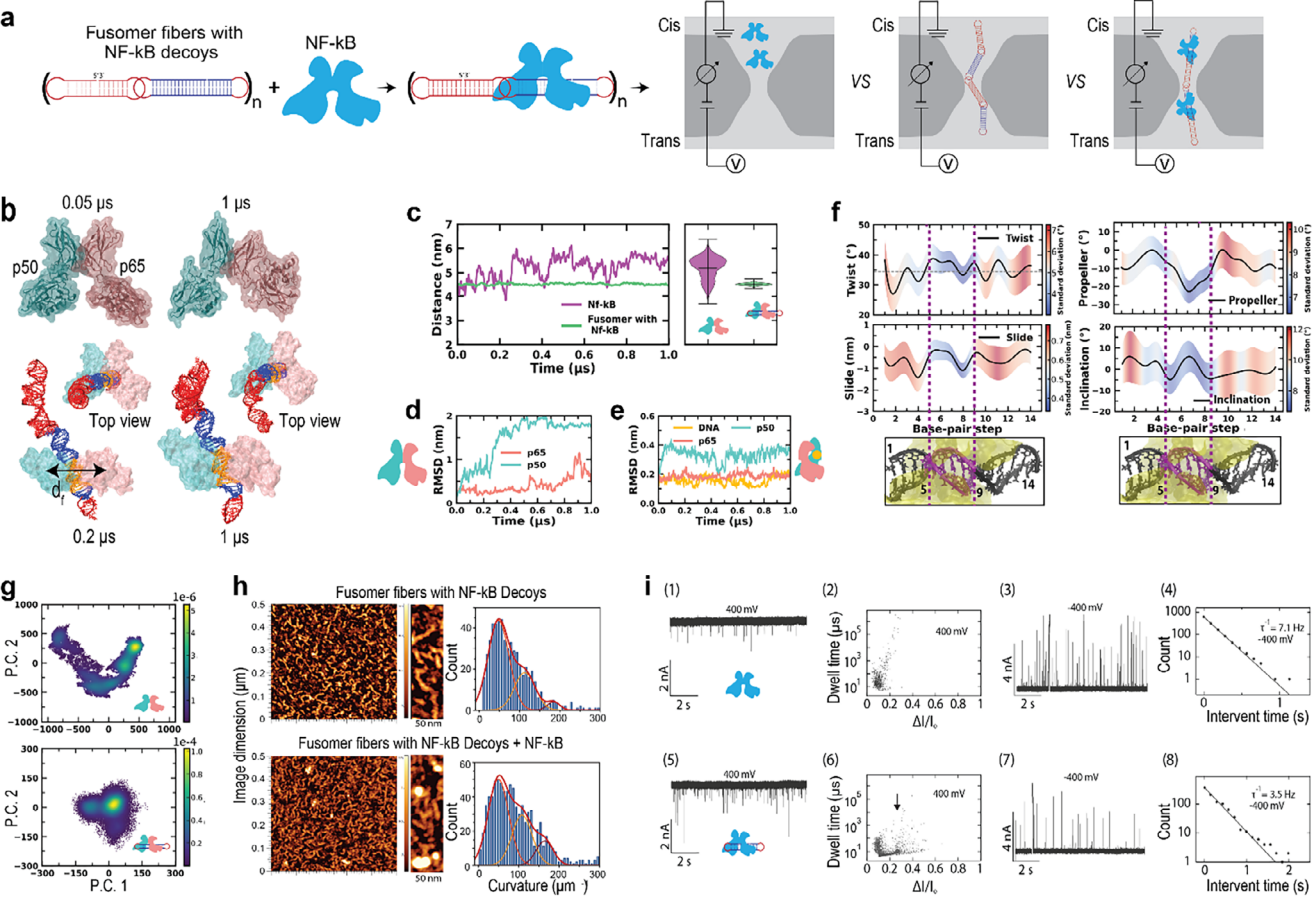
Nanopore‐assisted detection of NF‐κB protein using fusomer fibers functionalized with NF‐κB decoys. (a) Schematic of fusomer fibers functionalized with NF‐κB decoys, their binding to NF‐κB protein and nanopores analysis. (b) MD snapshots of NF‐κB and fusomer with NF‐κB decoy. (c) Distance between the center‐of‐mass of p50 and p65 subunits of NF‐κB (in free state as well as fusomer fiber‐bound state) as a function of time recorded during the MD simulation, (right) violin plots of the same. (d) RMSD of the two protein subunits (each individually aligned to the respective crystal structure) in the unbound state and (e) RMSD of p50, p65, and 6 bp dsDNA subunit of the fusomer encapsulated by NF‐κB (each individually aligned to the respective crystal structure). Data in (c), (d), and (e) are smoothed over a 0.5 ns window. (f) Twist, slide, propeller, and inclination of a 15 bp dsDNA subunit of the fusomer averaged over the MD trajectory. (g) PCA of Cartesian coordinates of non‐H atoms of NF‐κB in unbound state (top) and bound (to fusomer) state (bottom). (h) AFM topography images of fusomer fibers and fusomer fibers bound to NF‐κB protein. (i) Characterization of NF‐κB protein and its complex with NF‐κB decoy fusomer: (1) current trace and (2) scatter plot of fractional current blockade and dwell time at 400 mV, (3) current trace and (4) histogram of interevent time at −400 mV for NF‐κB protein. (5) Current trace and (6) scatter plot of fractional current blockade and dwell time at 400 mV (arrow shows the extra population of duplex‐bound protein), (7) current trace and (8) histogram of interevent time at −400 mV for the complex of NF‐κB protein with NF‐κB decoy duplex monomer. The concentration of the protein in each sample was 71 nm. Current traces were recorded at a sampling rate of 4167 kHz and low pass filtered at 250 kHz.

Fusomer fibers functionalized with embedded NF‐κB decoy sequences were analyzed for their ability to selectively bind the NF‐κB protein, yielding a distinctive signature as the complex translocated through nanopores (Figure 6a). Subsequently, all‐atom MD simulations were employed to evaluate the conformational flexibility of NF‐κB bound to the decoy‐functionalized fusomer fibers. This is essential for nanopore sensing, as the stability of the protein is crucial. Stable, uniform protein structures lead to more consistent, interpretable ionic current signals, improving sensitivity and accuracy of detection.

In the absence of fusomer fibers, the two NF‐κB subunits (p50 and p65) do not retain their structure and exhibit claw‐like motions (Figure 6b), with the binding cavity gradually opening and closing. This behavior was analyzed by measuring the distance between the centers of mass (COM) of the p50 and p65 subunits (Figure 6c). In the absence of DNA, the inter‐subunit distance fluctuated widely, averaging ∼5.2 nm with a standard deviation of ± 1 nm, indicating high flexibility and reduced structural stability. In contrast, the DNA‐bound protein maintained a shorter and more consistent inter‐subunit distance of ∼4.5 nm, suggesting a more constrained and stable configuration. Both p50 and p65 NF‐κB subunits, when unbound to DNA, had RMSD values exceeding 0.5 nm (Figure 6d), suggestive of structural deviation and dynamic flexibility. However, upon DNA binding, the RMSD (Figure 6e) values dropped below 0.35 nm, indicating that both subunits retain a well‐ordered structure over the course of the 1 µs simulation. The 6 bp DNA segment within the binding site also remained stable with an RMSD under 0.35 nm. Principal component analysis (Figure 6g) showed that unbound NF‐κB has a broad range of conformations, while the DNA‐bound state exhibits a tightly clustered distribution. This indicates that DNA binding stabilizes NF‐κB and restricts its internal dynamics. The dynamics of the DNA segment bound within the NF‐κB binding cavity differed notably from those of the adjacent unbound helix (Figure 6f). The bound DNA region showed a reduction in propeller angle and a relatively elevated inclination angle. Also, the bound DNA displayed a more restricted motion, as reflected by lower standard deviations in twist, slide, propeller, and inclination, angles compared to the unbound segment, and NF‐κB binding suppressed the local conformational flexibility of DNA within the binding pocket.

The RMSF fluctuates around 6 Å across all three fibers, with higher flexibility localized to the open loop regions. A similar trend is observed for the fusomer fibers when bound to NF‐κB, although with a lower overall RMSF of approximately 4 Å. In contrast, NF‐κB exhibits the most pronounced stabilization: upon forming hydrogen bonds with DNA, the protein largely retains its crystallographic conformation, resulting in substantially reduced fluctuations. The protein only binds to about 5 bp DNA domain of the fusomer fiber but still retains ∼7‐8 hydrogen bonds with the DNA throughout the simulation (Figure S11).

**FIGURE 7:**
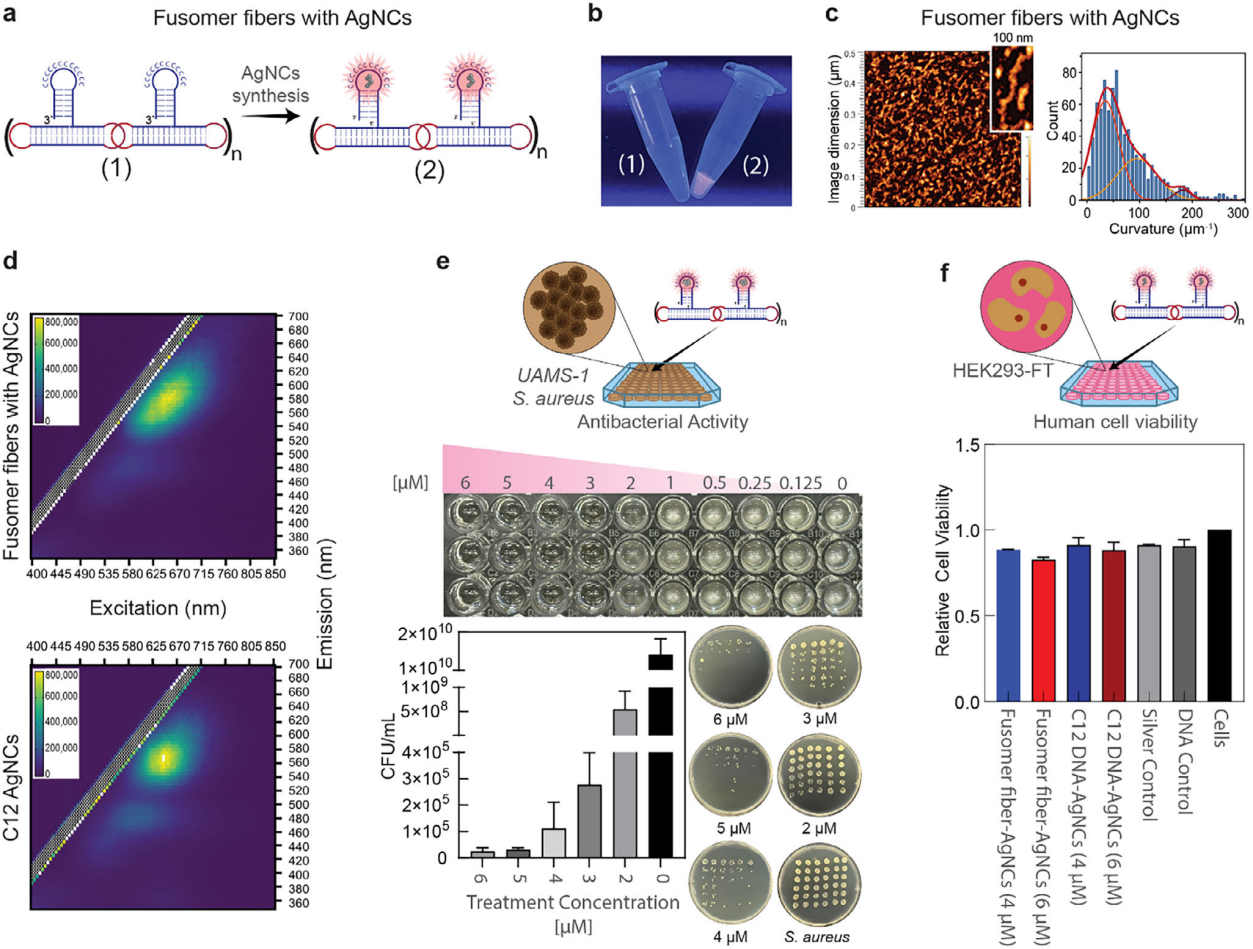
The synthesis, characterization, and assessment of antibacterial activity of fusomer fibers with AgNCs. (a) Synthesis of fusomer fibers with AgNCs. (b) Change of fluorescence of fusomer fibers upon fusomer fibers with AgNCs synthesis. (c) AFM image of the fusomer fibers with AgNCs. (d) EEM micrographs showing the fluorescence of fusomer fibers with AgNCs compared to C12 AgNCs. (e) Evaluating the antibacterial activity of fusomer fibers functionalized with AgNCs against UAMS‐1 S. aureus. Top: 96‐well plate showing the minimum inhibitory concentration (MIC) based on the absence of turbidity. Bottom left: Bar graph depicting CFU/mL of *S. aureus* with and without fusomer fibers with AgNCs treatment. Bottom right: Representative LB agar plates used to enumerate colonies and calculate CFU/mL. (f) HEK293‐FT cell viability measured by MTS assay upon treatment with fusomer fibers‐AgNCs (4–6 µm), carbenicillin, buffer, and DNA control. Relative cell viability is quantified and presented as mean ± SD, (*n* = 3).

To support the results from the MD simulations, fusomer fibers functionalized with NF‐κB decoys were analyzed by AFM (Figure 6h) and tested as molecular sensing probes for NF‐κB protein via nanopore analysis, shown in Figure 6i [100, 101]. The current trace recorded at 400 mV for the NF‐κB protein in 1 m KCl, 10 mm HEPES, 2 mm MgCl_2_ at pH 7.5 with a ∼5.2 nm pore is shown in Figure 6i‐1. The protein was slightly negatively charged (−4.61) in a folded state at pH 7.5 as calculated by PROPKA [102, 103]. A weakly negatively charged protein experiences a weak electrophoretic force supporting translocation and a strong electroosmotic force opposing translocation; therefore, shallow events were observed at positive voltages as shown in Figure 6i‐1,2. At −400mV, a strong electroosmotic force, which supports translocation, overcomes the weak electrophoretic force, thereby allowing the translocation. Consequently, deeper events were generated when a negative voltage was applied, as shown in Figure 6i‐3. In Figure 6i‐4, the histogram of interevent time is shown, where the capture rate is given by the exponential fit. The complex was prepared by mixing protein and fusomer monomers at a 1:2 ratio. When tested at 400 mV, the complex produced a population of deeper events (Figure 6i‐5,6), while such a population was absent for the protein. The current trace recorded at 400 mV for the complex with another pore also showed the deeper events, which were absent in the current trace of the monomer and protein as shown in Figure S12. This indicates the successful binding of the monomer to the NF‐κB protein. The binding increased the negative charge of the complex, consequently increasing the strength of the electrophoretic force that facilitated translocation, leading to the production of deeper events. Events similar to those of free proteins were observed at −400 mV (Figure 6i‐7), although at half the capture rate, as shown in Figure 6i‐6, which also indicates that some proteins were bound to the monomer. The concentration of the protein in each sample was 71 nm. Protein‐bound fusomer fibers with NF‐κB decoy showed clogging‐like events, which were absent for the protein and fusomer fibers alone, as shown in Figure S13. Similar events were observed for protein‐bound fusomer fibers with NF‐κB decoys and DS RNA for the sample prepared by mixing 30% protein and 70% fusomer fibers (Figure S14). Those events may indicate the successful binding of the protein with the fusomer fibers.

### 3.10 Fusomer Fibers Can be Designed for Potent Antibacterial Activity

Antibiotic resistance is a global health crisis and has been linked to millions of deaths worldwide [104]. To combat antibacterial resistance, nano‐silver is a novel, emerging antibacterial agent [105]. Specifically, DNA‐templated and stabilized silver nanoclusters (AgNCs) have recently been found to inhibit the growth of or kill multiple bacterial strains [21, 106, 107]. In this study, AgNCs templated with a C12 hairpin loop were tested alone and as a therapeutic moiety that was added to the fusomer fibers, forming fusomer fibers with AgNCs that fluoresce in the red‐spectra (Figure 7a,b). The structure of the fusomer fibers with the C12 hairpin is shown in the AFM topography image in Figure 7c. Excitation‐emission spectroscopy (EEM) of each AgNC sample was used to quantify the fluorescence (Figure 7d). Both C12 AgNCs and fusomer fibers with AgNCs emission peaks occurred within a visible orange‐red color. Notably, fusomer fibers with AgNCs had a broader emission spectrum, as compared to the small circular peak from the C12 AgNCs. Interestingly, C12 AgNCs had a higher emission intensity than fusomer fibers with AgNCs, which could be due to the localization of the C12 AgNCs emission. The known EEM spectra of the fusomer fibers with AgNCs, together with the highly modular structure of fusomer fibers, allow for the addition of targeting moieties for specific bacterial strains. In addition, such EEM spectra can permit the future use of fusomer fibers as biosensing and bioimaging tools. The potential use of fusomer fiber with AgNCs as biosensing molecules is further enhanced due to the fact that both the fusomer fibers with AgNCs and C12AgNCs emit in the red‐light range, which can penetrate 4–5 mm beneath the surface of human skin [108].

The MIC and MBC of free C12 AgNCs were both 6 µm (Figure S15). In contrast, fusomer fibers with AgNCs exhibited both MIC and MBC at 3 µm, as shown in Figure 7e. The bactericidal versus bacteriostatic activity can be assessed by the MBC/MIC ratio, with values less than 4 indicating bactericidal action [109]. These results suggest that using fusomer fibers as a scaffold for AgNCs offers bactericidal activity while maintaining negligible cytotoxicity toward mammalian cells (Figure 7f).

### 3.11 Fusomer Fibers With Anticoagulant Activity

**FIGURE 8:**
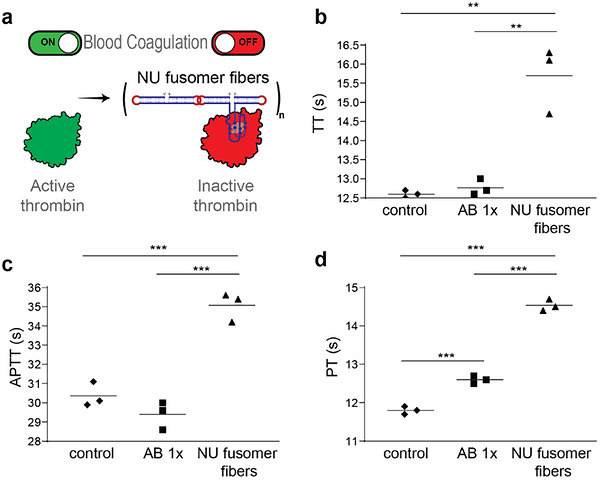
Antithrombin activity of NU fusomer fibers. (a) Schematic depiction of the anticoagulant effect of NU fusomer fibers. Plasma coagulation assessment of NU fusomer fibers using (b) Thrombin Time (TT), (c) Activated Partial Thromboplastin Time (APTT), and (d) Prothrombin Time (PT) tests with human plasma. The results depicted as average of (*n* = 3) biological repeats with three technical repeats each. Statistical significance according to the 1‐way ANOVA highlighted on the figure.

The coagulation system is central to hemostasis [110], promotes tissue repair [111], and interacts with the immune system [112]. When dysregulated, the coagulation system contributes to diseases such as thrombosis [113]. Anticoagulants are used to treat thrombosis; however, currently available treatments are limited by their side effects [114], narrow therapeutic window [115], and drug‐drug interactions [115, 116]. Hence, there is a need for new treatments that are safe and effective. DNA‐based aptamers with anticoagulant activity have been reported [117, 118]. The NU172 aptamer [118, 119, 120] is known for its anticoagulant activity, but its biomedical application has been hindered by its short half‐life. Therefore, there is a need for a system that can prolong the half‐life and enhance the stability of the NU172 aptamer. The versatility of fusomer fibers has enabled their use as a scaffold for the NU172 aptamers (NU fusomer fibers), to provide enhanced stability, increased local concentrations, and prolonged coagulation times (Figure 8a).

The PT, APTT, and TT tests are standard tests used in clinical practice to assess coagulation pathways in human plasma [118, 121]. APTT evaluates intrinsic pathway functionality, PT assesses the extrinsic pathway, and TT measures common pathway activity. These tests provide a comprehensive evaluation of the coagulation system, assisting in the diagnosis of bleeding disorders, evaluating hemostatic balance, and monitoring anticoagulant therapy.

To evaluate the anticoagulant potential of NU fusomer fibers, APTT, PT, and TT tests were performed using blood of healthy donors. Coagulation assays were conducted according to current clinical standards, using World Health Organization (WHO) certified human plasma as controls and WHO‐qualified plasma coagulation reagents with known time limits for normal plasma coagulation [122]. These standards set any time measurements below 11.5, 29, and 15.8 s as normal times for PT, APTT, and TT assays, respectively. Coagulation times above these limits were considered prolonged. Importantly, the coagulation times for all three assays were significantly prolonged by NU fusomer fibers (Figure 8b–d). To further demonstrate the advantage of NU fusomer fibers, free NU172 aptamers were tested for comparison. Various concentrations of free NU172 were evaluated using APTT, PT, and TT assays on plasma pooled from ten healthy donors. As shown in Figure S16, free NU172 at 0.5 µm did not alter coagulation parameters in any assay, whereas NU fusomer fibers at the same concentration produced clear effects. For free NU172, measurable changes were observed only at 1 µm. These findings are consistent with our previously established results [118].

### 3.12 Functionalization Affects the Mechanical Properties of Fusomer Fibers

AFM image analysis of functionalized fusomer fibers was performed and the results showed that functionalized fusomer fibers exhibit similar topological features to the non‐functionalized ones, with linear nanostructures showing curvature, indicating composition‐dependent flexibility. Generally, modifications led to increased flexibility, as demonstrated by a broader κ value distribution, as shown in the histograms in Figure S17. The presence of more flexible fibers was further evidenced by a larger contribution of Gaussians with higher κ values. The specific κ values obtained were as follows: F1‐15:F2‐15 fusomer fibers κ = 82.1 µm^−^^1^, fusomer fibers of dual function κ = 91.7 µm^−^^1^, fusomer fibers of dual function bound to NF‐κB protein κ = 107 µm^−^^1^, and fusomer fibers with C12 hairpin κ = 78.6 µm^−^^1^. Interestingly, the binding of the protein increases fiber flexibility, as indicated by the notable presence of higher κ values in the distribution.

## 4 Conclusion

In this study, we introduce a novel class of 1D hybrid RNA‐DNA fibrous NANPs, termed fusomer fibers, which combine the structural and functional properties of RNA and DNA in a chemically synthesized single sequence. Fusomer fibers offer a versatile and easily functionalized platform for diverse biomedical applications while remaining structurally tunable and cost‐effective. This study followed an integrated approach to comprehensively characterize the physicochemical, mechanical, immunological, and biological properties of fusomer fibers. Our results provide a comprehensive understanding of the behavior and functionality of fusomer fibers, demonstrating their potential for a wide range of biomedical applications and promise as next‐generation therapeutic agents. We have highlighted this potential by demonstrating multiple successful applications for these fusomer fibers in gene silencing, enzyme inhibition, protein downregulation, anti‐inflammatory function, bactericidal activity, and biosensing.

While the present study establishes the foundational design principles, assembly behavior, and functional adaptability of this new class of nanomaterials, successful in vivo intracellular delivery of our novel fusomers will require incorporation into an appropriate carrier system, such as polymeric [24, 73, 123], lipid‐based [124, 125, 126], or inorganic [127, 128] delivery agents, for intracellular delivery. Importantly, these carrier systems are also anticipated to protect fusomers from extracellular degradation, as previously demonstrated for other nucleic acid cargos [24, 73, 123, 124, 125, 126, 127, 128]. This will prolong circulation time and determine biodistribution and tissue targeting. As such, the development of fusomer‐carrier formulations will require rational carrier selection, validation of therapeutic cargo loading and cell‐based testing, prior to evaluation of delivery and efficacy in animal disease models, and assessment of off‐target effects and toxicity. In addition, the full therapeutic potential of fusomer fibers remains to be explored. Future efforts include the investigation of the use of fusomer fibers in targeted drug delivery and responsive nanodevices that integrate sensing and therapeutic functions. Together, these strategies will expand the horizon of nucleic acid nanotechnology and establish fusomer fibers as a potentially transformative, multifunctional platform for the development of new therapeutic interventions.

## Author Contributions

Y.R.: contributed to sequence design, synthesis of samples for experiments, reporter cell lines immunorecognition, GFP silencing, uptake in 3D organ‐on‐chips, dehydration experiments, serum stability experiment, data analysis, manuscript writing. L.P.R.: cGAS mechanistic study, GFP silencing, reporter cell lines immunorecognition experiments, cellular uptake mechanistic study, synthesis of samples for uptake in 3D organ‐on‐chips, data analysis. Y.I.A., M. R. C., and M.A. D.: experiments with PBMCs, data analysis. M.R.C.: uptake in 3D organ‐on‐chips, data analysis. E.S.: antibacterial activity, EEM, data analysis. L.R.: contributed to sequence design, tested initial hypothesis, sample preparation for AFM imaging and PBMCs analysis. J.N.H., A.J.L., and A.V.K.: AFM imaging, image analysis. R.F.S., T.K.F., A.L.L., and R.C.: immunofluorescence analysis, RT‐PCR for NF‐κB activation, antithrombin activity of fusomer fibers. H.C. and A.A.: MD simulations, data analysis. L.K.P. and M.W: nanopores experiments. I.M.: Provided critical insights into the manuscript, data analysis. K.A.A.: conceptualization, sequence design, project supervision, funding acquisition, data analysis, manuscript writing. All authors contributed to manuscript writing.

## Conflicts of Interest

The authors declare no conflicts of interest.

## Supporting information




**Supporting File**: smll74113‐sup‐0001‐SuppMat.docx

## Data Availability

The data that support the findings of this study are available from the corresponding author upon reasonable request.
