# Supplementary material for: RNA‐DNA Fusomer Fibers With Customizable Physicochemical, Mechanical, and Biological Properties for Next‐Generation Therapeutics
Source: Small. 2026 Jun 9;22(44):e00078. doi: 10.1002/smll.202600078 (PMC13324882; doi:10.1002/smll.202600078)
Supplement: Supplementary file 1 [file SMLL-22-e00078-s001.docx]

Supporting Information

**RNA-DNA Fusomer Fibers with Customizable Physicochemical, Mechanical, and Biological Properties for Next-Generation Therapeutics**

Yasmine Radwan^1^, Laura P. Rebolledo^1^, Yelixza I. Avila^1^, Elizabeth Skelly^1^, Lauren Rackley^1^, Julio Navas Hernandez^2^, Renata de Freitas Saito^3,4^, Laxmi K. Pandey^5^, Hemani Chhabra^6^, Alexander J. Lushnikov^2^, Tatiane Katsue Furuya^3,4^, Ana Luiza Lomba^3,4^, Da Shi^7^, Edward Cedrone^7^, Ian Marriott^8,9^, Morgan R. Chandler^10^, Meni Wanunu^5^, Aleksei Aksimentiev^6,11^, Alexey V. Krasnoslobodtsev^2^, Roger Chammas^3,4^, Marina A. Dobrovolskaia^7^, Kirill A. Afonin^1,9^*

^1^Chemistry and Nanoscale Science Program, Department of Chemistry, University of North Carolina Charlotte, Charlotte, NC 28223, USA

^2^Department of Physics, University of Nebraska Omaha, Omaha, NE 68182, USA

^3^Center for Translational Research in Oncology (LIM24), Instituto do Câncer do Estado de São Paulo (ICESP), Hospital das Clínicas da Faculdade de Medicina da Universidade de São Paulo (HCFMUSP), Av. Dr. Arnaldo, 251, 8th floor, São Paulo, SP, CEP 01246-000, Brazil

^4^Comprehensive Center for Precision Oncology, Universidade de São Paulo, Av. Dr. Arnaldo, 251, 8th floor, São Paulo, SP, CEP 01246-000, Brazil

^5^Department of Physics, Northeastern University, Boston, MA 02115, USA

^6^Beckman Institute for Advanced Science and Technology, University of Illinois at Urbana-Champaign, Urbana, IL 61801, USA

^7^Nanotechnology Characterization Laboratory, Cancer Research Technology Program, Frederick National Laboratory for Cancer Research sponsored by the National Cancer Institute, Frederick, MD 21701, USA

^8^Department of Biological Sciences, University of North Carolina at Charlotte, Charlotte, NC 28223, USA

^9^Center for Innovation, Translational Research and Applications of Nanostructured Systems (CITRANS), University of North Carolina at Charlotte, Charlotte, NC 28223, USA

^10^MIMETAS US, INC, Gaithersburg, MD 20878, USA

^11^Department of Physics, University of Illinois at Urbana-Champaign, Urbana, IL 61801, USA

* - correspondence to Kirill A. Afonin at kafonin@charlotte.edu

**Sequences Used in this project**

(RNA is red, DNA is blue, RNA kissing loops are in **bold**)

**Six-stranded RNA cube:**

1. 5'-rGrGrCrArArCrUrUrUrGrArUrCrCrCrUrCrGrGrUrUrUrArGrCrGrCrCrGrGrCrCrUrUrUrUrCrUrCrCrCrArCrArCrUrUrUrCrArCrG
2. 5'-rGrGrGrArArArUrUrUrCrGrUrGrGrUrArGrGrUrUrUrUrGrUrUrGrCrCrCrGrUrGrUrUrUrCrUrArCrGrArUrUrArCrUrUrUrGrGrUrC
3. 5'-rGrGrArCrArUrUrUrUrCrGrArGrArCrArGrCrArUrUrUrUrUrUrCrCrCrGrArCrCrUrUrUrGrCrGrGrArUrUrGrUrArUrUrUrUrArGrG
4. 5'-rGrGrCrGrCrUrUrUrUrGrArCrCrUrUrCrUrGrCrUrUrUrArUrGrUrCrCrCrCrUrArUrUrUrCrUrUrArArUrGrArCrUrUrUrUrGrGrCrC
5. 5'-rGrGrGrArGrArUrUrUrArGrUrCrArUrUrArArGrUrUrUrUrArCrArArUrCrCrGrCrUrUrUrGrUrArArUrCrGrUrArGrUrUrUrGrUrGrU
6. 5'-rGrGrGrArUrCrUrUrUrArCrCrUrArCrCrArCrGrUrUrUrUrGrCrUrGrUrCrUrCrGrUrUrUrGrCrArGrArArGrGrUrCrUrUrUrCrCrGrA

**Six-stranded DNA cube:**

1. 5'-GGCAACTTTGATCCCTCGGTTTAGCGCCGGCCTTTTCTCCCACACTTTCACG
2. 5'-GGGAAATTTCGTGGTAGGTTTTGTTGCCCGTGTTTCTACGATTACTTTGGTC
3. 5'-GGACATTTTCGAGACAGCATTTTTTCCCGACCTTTGCGGATTGTATTTTAGG
4. 5'-GGCGCTTTTGACCTTCTGCTTTATGTCCCCTATTTCTTAATGACTTTTGGCC
5. 5'-GGGAGATTTAGTCATTAAGTTTTACAATCCGCTTTGTAATCGTAGTTTGTGT
6. 5'-GGGATCTTTACCTACCACGTTTTGCTGTCTCGTTTGCAGAAGGTCTTTCCGA

**RNA Fiber:**

1. 5'-rGrGrGrArArUrCrC**rArArGrGrArGrGrCrA**rGrGrArUrUrCrCrCrGrUrCrArCrArG**rArArGrGrArGrGrCrA**rCrUrGrUrGrArC
2. 5'-rGrGrGrArArCrGrU**rArArGrCrCrUrCrCrAr**ArCrGrUrUrCrCrCrGrGrArUrGrCrUr**ArArGrCrCrUrCrCrA**rArGrCrArUrCrC

**Fusomer fibers fiber (8 bp):**

1. 5'- GGGA**rArArGrGrArGrGrCrA**TCCCGCCC**rArArGrGrArGrGrCrA**GGGC
2. 5'- CGC**rArArGrCrCrUrCrCrA**GCGTTCGC**rArArGrCrCrUrCrCrA**GCGAA

**Fusomer fibers fiber (15 bp):**

1. 5'-GGGAATCCrArArGrGrArGrGrCrAGGATTCCCGTCACAGrArArGrGrArGrGrCrACTGTGAC
2. 5'-GGGAACGT**rArArGrCrCrUrCrCrA**ACGTTCCCGGATGCT**rArArGrCrCrUrCrCrA**AGCATCC

**Fusomer fibers fiber (30 bp):**

1. 5'-GGGAAAGGAAGGACT**rArArGrGrArGrGrCrA**AGTCCTTCCTTTCCCCGCGTATAGT CACAG**rArArGrGrArGrGrCrA**CTGTGACTATACGCG
2. 5'-GGGAAGGAAGGAAGA**rArArGrCrCrUrCrCrA**TCTTCCTTCCTTCCCCGATGCTCTA CTAGG**rArArGrCrCrUrCrCrA**CCTAGTAGAGCATCG

**RNA-Fusomer (15 bp) fiber:**

1. 5'-rGrGrGrArArUrCrC**rArArGrGrArGrGrCrA**rGrGrArUrUrCrCrCrGrUrCrArCrArG**rArArGrGrArGrGrCrA**rCrUrGrUrGrArC
1. 5'-GGGAACGT**rArArGrCrCrUrCrCrA**ACGTTCCCGGATGCT**rArArGrCrCrUrCrCrA**AGCATCC

**Fusomer (15 bp)-RNA fiber:**

1. 5'-GGGAATCC**rArArGrGrArGrGrCrA**GGATTCCCGTCACAG**rArArGrGrArGrGrCrA**CTGTGAC
2. 5'-rGrGrGrArArCrGrU**rArArGrCrCrUrCrCrA**rArCrGrUrUrCrCrCrGrGrArUrGrCrU**rArArGrCrCrUrCrCrA**rArGrCrArUrCrC

**Fusomer (15 bp)-Fusomer (30 bp) fibers:**

1. 5'-GGGAATCC**rArArGrGrArGrGrCrA**GGATTCCCGTCACAG**rArArGrGrArGrGrCrA**CTGTGAC
2. 5'-GGGAAGGAAGGAAGA**rArArGrCrCrUrCrCrA**TCTTCCTTCCTTCCCCGATGCTCT ACTAGG**rArArGrCrCrUrCrCrA**CCTAGTAGAGCATCG

**Fusomer (30 bp)-Fusomer (15 bp) fibers:**

1. 5'-GGGAAAGGAAGGACT**rArArGrGrArGrGrCrA**AGTCCTTCCTTTCCCCGCGTATAGT CACAG**rArArGrGrArGrGrCrA**CTGTGACTATACGCG
2. 5'-GGGAACGT**rArArGrCrCrUrCrCrA**ACGTTCCCGGATGCT**rArArGrCrCrUrCrCrA**AGCATCC

**Fusomer (8 bp)-Fusomer (15 bp) fibers:**

1. 5'- GGGA**rArArGrGrArGrGrCrA**TCCCGCCC**rArArGrGrArGrGrCrA**GGGC
2. 5'-GGGAACGT**rArArGrCrCrUrCrCrA**ACGTTCCCGGATGCT**rArArGrCrCrUrCrCrA**AGCATCC

**Fusomer (8 bp)-Fusomer (30bp) fibers:**

1. 5'- GGGA**rArArGrGrArGrGrCrA**TCCCGCCC**rArArGrGrArGrGrCrA**GGGC
2. 5'-GGGAAGGAAGGAAGA**rArArGrCrCrUrCrCrA**TCTTCCTTCCTTCCCCGATGCTCTACTAGG**rArArGrCrCrUrCrCrA**CCT AGTAGAGCATCG

**RNA-Fusomer fibers with embedded NF-kB Decoy:**

1. 5'-GAGGGAAATCCCTTC**rArArGrGrArGrGrCrA**GAAGGGATTTCCCTCGUCACAG**rArArGrGrArGrGrCrA**CUGUGAC
2. 5'-rGrGrGrArArCrGrU**rArArGrCrCrUrCrCrA**rArCrGrUrUrCrCrCrGrGrArUrGrCrU**rArArGrCrCrUrCrCrA**rArGrCrArUrCrC

**Fusomer fibers with embedded NF-kB Decoy:**

1. 5'-GAGGGAAATCCCTTC**rArArGrGrArGrGrCrA**GAAGGGATTTCCCTCGUCACAG**rArArGrGrArGrGrCrA**CUGUGAC
2. 5'-GGGAACGT**rArArGrCrCrUrCrCrA**ACGTTCCCGGATGCT**rArArGrCrCrUrCrCrA**AGCATCC

**Fusomer-RNA fibers of dual function (with embedded NF-kB Decoy and appended DS RNA against NF-Kb):**

1. 5'-GAGGGAAATCCCTTC**rArArGrGrArGrGrCrA**GAAGGGATTTCCCTCGUCACAG**rArArGrGrArGrGrCrA**CUGUGAC
2. 5'- rGrGrGrArArCrGrU**rArArGrCrCrUrCrCrAr**ArCrGrUrUrCrCrCrGrGrArUrGrCrU**rArArGrCrCrUrCrCrA**rArGrCrArUrCrC rUrUrCrGrUrArArArArGrGrArCrArUrArUrGrArGrArCrCrUrUrCrArArUrU
3. /5Phos/rUrUrGrArArGrGrUrCrUrCrArUrArUrGrUrCrCrUrUrUrUrArCrG

**NF-kB Decoy Duplex:**

1. 5'-CCTTGAAGGGATTTCCCTCC
2. 5'-GGAGGGAAATCCCTTCAAGG

**RNA fibers with one strand labelled with IRD800:**

1. 5'-rGrGrGrArArUrCrC**rArArGGrArGrGrCrA**rGrGrArUrUrCrCrCrGrUrCrArCrAG**rArArG rGrArGrGrCrA**rCrUrGrUrGrArC
2. /5IRD800/rGrGrGrArArCrGrU**rArArGrCrCrUrCrCrA**rArCrGrUrUrCrCrCrGrGrArUrGrCrU**rArArGrCrCrUrCrCrA**rArGrCrArUrCrC

**Fusomer fibers with one strand labelled with IRD800:**

1. 5'-GGGAATCC**rArArGrGrArGrGrCrA**GGATTCCCGTCACAG**rArArGrGrArGrGrCrA**CT GTGAC
2. /5IRD800/GGGAACGT**rArArGrCrCrUrCrCrA**ACGTTCCCGGATGCT**rArArGrCrCrUrCrCrA**AGCATCC

**Fusomer fibers with AgNCs:**

1. 5'-GGGAATCC**rArArGrGrArGrGrCrA**GGATTCCCGTCACAG**rArArGrGrArGrGrCrA** CTGTGACTATCCGTCCCCCCCCCCCCACGGATA
2. 5'-GGGAACGT**rArArGrCrCrUrCrCrA**ACGTTCCCGGATGCT**rArArGrCrCrUrCrCrA** AGCATCCTATCCGTCCCCCCCCCCCCACGGATA

**C12 hairpin template for AgNCs:**

1. 5'-TATCCGTCCCCCCCCCCCCACGGATA

**Fusomer fibers with antithrombin aptamer (NU172):**

1. 5'-GGGAATCC**rArArGrGrArGrGrCrA**GGATTCCCGTCACAG**rArArGrGrArGrGrCrA**CT GTGAC
2. 5'-GGGAACGT**rArArGrCrCrUrCrCrA**ACGTTCCCCGCCTAGGTTGGGTAGGGTGGTG GCGGGATGCT**rArArGrCrCrUrCrCrA**AGCATCC

**Fusomer-RNA fibers appended with** **DS RNA against GFP:**

1. 5'-rGrGrGrArArUrCrC**rArArGrGrArGrGrCrA**rGrGrArUrUrCrCrCrGrUrCrArCrArG**rArArG rGrArGrGrCrA**rCrUrGrUrGrArCrUrUrUrGrGrUrGrGrUrGrCrArGrArUrGrArArCrUrUrCrArGrGrGrUrCrA
2. 5'-GGGAACGT**rArArGrCrCrUrCrCrA**ACGTTCCCGGATGCT**rArArGrCrCrUrCrCrA**AG CATCC
3. 5'-prArCrCrCrUrGrArArGrUrUrCrArUrCrUrGrCrArCrCrArCrCrG

**DS RNA against GFP:**

1. 5'-prArCrCrCrUrGrArArGrUrUrCrArUrCrUrGrCrArCrCrArCrCrG
2. 5'-rCrGrGrUrGrGrUrGrCrArGrArUrGrArArCrUrUrCrArGrGrGrUrCrA

**Fusomer-RNA fiber with DS against GFP labelled with A488:**

1. 5'-GGGAATCC**rArArGrGrArGrGrCrA**GGATTCCCGTCACAG**rArArGrGrArGrGrCrA**CT GTGAC
2. 5'-rGrGrGrArArCrGrU**rArArGrCrCrUrCrCrA**rArCrGrUrUrCrCrCrGrGrArUrGrCrU**rArArG**

**rCrCrUrCrCrA**rArGrCrArUrCrCrUrUrUrGrGrUrGrGrUrGrCrArGrArUrGrArArCrUrUrCrArGrGrGrUrCrA

3. 5'-TGACCCTGAAGTTCATCTGCACCACCG/AlexF488/

**EXTENDED MATERIALS AND METHODS**

*Design and production of fusomer fibers*

*Design of RNA-DNA fusomer fibers:* The RNA-DNA monomers fold into a dumbbell shape comprised of 9-nucleotide (nt) long ssRNA interacting motifs (kissing loops) on either side of the dsDNA stem, which can vary in length and carry specific functionalities.

In general, all self-assembling fiber structures were composed of 2 monomers per repeat unit that specifically interact together *via* the kissing loops. This provided the ability to design multifunctional fusomer fibers, where each monomer has its own distinct functionality. The nomenclature used for the non-functionalized fibers throughout the manuscript indicates if monomer 1 is R1 = RNA or F1 = fusomer, and monomer 2 is R2 = RNA or F2 = fusomer, the number following each monomer indicates the number of base pairs (bps) in the stem of the monomer, for example F1-8 means the monomer 1 is a fusomer with 8 bps in the dsDNA stem. For ease, fibers containing both fusomer monomers will be referred to as fusomer fibers.

Three different stem lengths were designed: fusomer fibers with 8, 15, and 30-bp dsDNA stems. In addition, fusomer fibers with embedded NF-κB decoy sequences were designed for NF-κB protein binding and attenuation of its function or its biosensing with nanopores. RNA-fusomer fibers were designed for gene silencing *via* RNA interference (RNAi) by functionalizing individual RNA monomers with Dicer-substrate (DS) RNAs targeting either NF-κB or GFP. Fusomer fibers with C12 hairpins were used to template silver nanoclusters (AgNCs) for antibacterial activity. Finally, fusomer fibers with thrombin binding NU 172 aptamers were designed for anticoagulant activity.

*Production and storage of fibers and fusomer fibers:* RNA monomers were synthesized by *in vitro* transcription (IVT). Corresponding DNA templates were amplified by PCR using MyTaq Mix and purified with the DNA Clean & Concentrator kit (Zymo Research). Run-off IVT was performed using T7 RNA polymerase in buffer containing 80 mM HEPES-KOH (pH 7.5), 50 mM DTT, 25 mM MgCl₂, 2.5 mM spermidine, and 5 mM of each rNTP at 37°C for 3.5 hr. Transcription was terminated by the addition of RQ1 RNase-Free DNase (Promega), followed by incubation at 37°C for 30 minutes to degrade DNA templates. RNA products were purified by denaturing polyacrylamide gel electrophoresis (PAGE; 8% acrylamide, 8 M urea) run at 13 W for 2 hr in 1X TBE buffer (89 mM Tris–borate, 2 mM EDTA, pH 8.2). RNA bands were visualized by UV shadowing, excised, and eluted overnight at 4°C in TBE buffer (pH 8.2) containing 300 mM NaCl. Eluted RNA was precipitated by the addition of 2.5 volumes of 100% ethanol and incubation at −20°C for 4 hours, followed by centrifugation at 10,000 X G for 30 minutes at 4°C. Pellets were washed twice with 90% ethanol by centrifugation at 10,000 X G for 10 minutes at 4°C. RNA pellets were vacuum-dried using a CentriVap micro-IR concentrator (Labconco) at 55°C and resuspended in HyPure cell culture-grade water (Invitrogen). RNA concentrations were determined by UV absorbance at 260 nm using a NanoDrop 2000 spectrophotometer (Thermo Fisher Scientific), and samples were stored at -20°C.

For assembly of all fibers, corresponding monomer strands were combined in equimolar ratios with HyPure cell culture-grade water and assembled in a one-pot thermal anneal, by heating to 95°C for 2 min, snap-cooling on ice for 2 min, then adding the assembly buffer (89 mM tris-borate (pH 8.2), 2 mM MgCl_2_, 50 mM KCl), and incubating for 20 min at room temperature (RT). All fibers were stored at 4°C until use.

Control RNA and DNA cubes were assembled by mixing six cognate strands at equimolar ratios, heating at 95°C for 2 minutes, followed by incubation at 45°C for 2 minutes. Assembly buffer was then added and samples were incubated for an additional 30 minutes at 45°C.

To confirm successful assembly of all fibers, 8% native-PAGE (37.5:1 acrylamide:bis-acrylamide) was used for visualization. A Mini-PROTEAN Tetra Cell system (Bio-Rad) was used to prepare the gel, then the gel was pre-run at 150 V for 10 min with running buffer (89 mM TB (pH 8.2), and 2 mM MgCl_2_). To load samples, 2 µL of loading buffer (assembly buffer, 30% glycerol, bromophenol blue, xylene cyanol), was mixed with 2 µL of each fiber sample, and 4 µL were loaded per well. The gel was run for 30 minutes at 300 V at 4°C. Ethidium bromide solution (EtBr, 0.5 µg mL^-1^_,_ VWR Chemicals) was used to stain the gel for 5 minutes, then the gel was washed twice with double-deionized water (ddiH_2_O). ChemiDoc MP (Bio-Rad) was used to image the gel.

For AgNCs synthesis (Fusomer fibers with AgNCs), 75 μM of fusomer fibers-AgNCs assembled with assembly buffer (89 mM tris-borate (pH 8.2), 2 mM MgCl_2_), HyPure cell culture-grade water, 1 mM AgNO_3_, and 20 mM NH_4_OAc (pH 6.9) were combined, mixed, and centrifuged, then incubated at 95°C for 2 minutes. Following that, the solutions were incubated on ice for 20 min. 10 mM NaBH_4_ solution was prepared fresh using chilled water and kept on ice. Equimolar concentration of NaBH_4_ to Ag^+^ in the fusomer fibers-AgNCs samples was used to reduce the samples. Control samples containing C12 DNA-AgNCs were prepared by replacing fusomer fibers with C12HP DNA, while a silver control solution was prepared by replacing the fusomer fibers volume with HyPure cell culture-grade water. All samples were stored in the dark at 4°C for ~16 hours(1, 2). The synthesis of DNA- and fusomer fibers-AgNCs was confirmed by visualizing the fluorescence of the samples under UV light (340 nm).

*Dehydration and stability:* For fusomer fibers long term storage and for shipping, the stability was assessed upon dehydration and rehydration following an established protocol(3) with minor modifications. Two dehydration methods were tested: lyophilization and vacuum concentration. RNA fibers, fusomer fibers with 15-bps dsDNA stem, and fusomers fibers with embedded NF-κB decoy samples (all at 1 µM), were aliquoted (5 µL per tube). For the lyophilization method, a VirTis SP Scientific Benchtop Pro with an Omnitronics freeze dryer was used. The samples were frozen for 3 minutes in liquid nitrogen before lyophilizing at 20.5°C shelf temperature, a ∼−91°C condenser, and ∼20 mTorr vacuum overnight. The samples were collected and equilibrated with atmospheric air, then sealed with parafilm. For the vacuum concentration method, a CentriVap micro-IR vacuum concentrator (Labconco, Speedvac) was used. The samples were centrifuged at 60°C until fully dry then sealed with parafilm. All dehydrated samples were kept in a heat block for one week at 55°C. As a control, samples were kept in solution at 4°C and 55°C for one week. After one week, the dehydrated samples were rehydrated with ddH_2_O (5 µL), mixed gently by pipetting, and centrifuged. Rehydrated samples and all control samples were visualized on a native-PAGE or kept in the cold room at 4°C until needed. To assess stability, an 8% (37.5:1) polyacrylamide native-PAGE was run in running buffer (89 mm tris-borate (pH 8.2) and 2 mM MgCl_2_) at 300 V for 20 minutes in a cold room (4°C). Total EtBr staining was done for 5 min before imaging the gel using a ChemiDoc MP system (Bio-Rad).

*Physicochemical characterization*

*AFM imaging**:* Freshly cleaved mica was immersed in 167 µM aqueous solution of 1-(3-aminopropyl)-silatrane (APS) for 30 minutes(4, 5). The APS modified mica pieces were rinsed in deionized water, dried under a stream of ultra-high purity argon (AR UHP), and further dried under vacuum for at least 12 hours. RNA fibers and fusomer fibers samples were diluted at 4°C with assembly buffer and immediately deposited onto APS-modified mica for 2 minutes from the buffered solution, rinsed briefly with deionized water, and dried with a gentle flow of argon(6, 7). Various concentrations were deposited and imaged. This was necessary to achieve desired coverage and to avoid, as much as possible, fiber nanostructures overlapping for image analysis of curvature, end to end distance, R_EE_, and contour length, L_C_.

Imaging was performed with a MultiMode AFM Nanoscope IV system (Bruker Instruments, Santa Barbara, CA, USA) in Tapping Mode at ambient conditions. The images were recorded with a scanning rate of 1.5 Hz using an RTESPA-300 probe (Bruker Nano Inc., CA, USA) with a resonance frequency of ~320 kHz and a spring constant of ~40 N*/*m. Images were processed using the FemtoScan Online software(6, 7).

Analysis of curvature of the resultant nanostructures was done using a “Kappa” plugin with the Fiji software(8). First, the length scale of each image was calibrated from pixel to nm. Then, each fiber nanostructure was manually traced with an open B-spline curve. “Kappa” automatically fitted the curve to the topology of individual fibers and returned the length of each fiber and the values of curvature at every point on the curve. Custom home-written Python code was used to calculate the end-to-end distance by identifying the starting and ending points of a fiber. Statistical histograms of the data points were plotted with MagicPlot 3.0 Pro software and further fitted by Gaussian functions.

*Molecular dynamics*

*Coarse-grained simulations of RNA polymers:* Coarse-grained RNA-RNA molecular dynamics (MD) simulations were performed using the sequence-dependent oxRNA2 model(9, 10). oxRNA2 is a nucleotide-level coarse-grain RNA model designed to reproduce structural, mechanical, and thermodynamic properties of RNA. Monomeric RNA strands R1 and R2 (R1-15 bp and R2-15 bp have different sequences) were constructed using oxview(11). Each strand was minimized for 20,000 steps followed by a subsequent relaxation for 3.06 ns, employing an Andersen-like john thermostat at 310 K (37°C) and 1 M monovalent salt using a 15-fs integration timestep.

*System setup:* To study the behavior of different RNA lengths, we assembled n = 1 RNA polymer by placing R1 and R2 (where R1 and R2 are monomeric units of the RNA fusomer that form a kissing loop interaction) in close vicinity such that the kissing interaction forming loops face each other. Harmonic traps with a force constant of 57.08 pN/nm were introduced between the complementary bases of the kissing interaction forming loops of RNA strands, facilitating the formation of kissing loops. A short NVT simulation of 30 ns with harmonic traps was then carried out to enable the formation of kissing loops using the GPU implementation of oxRNA2.

RNA polymers with n = 2, n = 4, n = 8 and n = 16 were assembled by duplicating n = 1, n = 2, n = 4 and n = 8 RNA polymers, respectively, in oxview and forcing formation of kissing loops using harmonic traps. The final configurations of long strands with fully formed kissing loops were simulated for 9 µs without any external forces. This extended MD simulation aimed to capture the dynamics and length-dependent properties of RNA polymers.

*All-atom MD simulations:* All MD simulations were performed using NAMD2.14(12) using 2 fs integration timestep and 2-2-6 multiple time stepping. Bsc1, OL3 and ffSB14 parameters were used for DNA(13), RNA(14) and proteins(15) respectively, TIP3P for water(16), with CUFIX corrections to model interactions between ions and nucleic acid/proteins(17). The SETTLE algorithm(18) was employed to enforce the rigidity of covalently bonded hydrogen atoms within water molecules. Additionally, for non-water molecules, the RATTLE algorithm(19) was utilized to constrain the motion of hydrogen atoms involved in covalent bonds. The long-range electrostatic interactions were computed using the particle mesh Ewald (PME) scheme over a 1-Å-spaced grid(20) and periodic boundary conditions (PBC). Van der Waals (VDW) and short-range electrostatic forces were evaluated using the 10–12 Å smooth cut-off scheme. Non-bonded interactions were implemented using “1-4 scaling” with a scaling factor of 0.833.

NPT (constant number of particles N, pressure P and temperature T) simulations(21) were performed using a Nosé-Hoover Langevin piston with a period of 400 fs, decay of 200 fs at a target pressure of 1 atm and Langevin thermostat(22) set at 310 K with a 0.5 ${ps}^{-1}$ damping. Simulations performed in the NVT (constant number of particles *N,* volume *V* and temperature *T)* ensemble employed the Langevin thermostat. Energy minimization was carried out using conjugate gradients(23). Water and ions were added to produce a neutral system with 1 M KCl. Atomic coordinates were saved every 18 ps. VMD(24) and MDAnalysis(25) were used for visualization and analysis.

Each system was minimized for 20,000 steps and equilibrated in NPT ensemble for 4.2 ns. During minimization and NPT equilibration, harmonic restraints with a spring constant of 5 kcal/(mol$Å^{2})$ were applied to non-hydrogen atoms of nucleic acid and protein (NF-kB). The production simulations, each spanning 1 μs, were conducted within an NVT ensemble, devoid of any restraints.

*All-atom system setup:* The R1-R2 all-atom representation for n = 1 was obtained by converting the equilibrated R1-R2 oxRNA2 structure using a custom built mrDNA(26) script. All-atom representation of RNA-fusomer fibers (R1-15 bp and F2-15bp) and fusomer-fusomer fibers (F1-15bp and F2-15bp) were constructed using oxview and then converted to all-atom representations using tacoxDNA(27), ensuring the A- and B-forms for RNA and DNA, respectively, were conserved. For the NF-kB simulation focusing solely on the protein (PDB ID: 1VKX), DNA resolved in the structure was removed from the protein cavity. Subsequently, the protein was protonated using H++(28) corresponding to a pH of 7.5. The NF-kB-fusomer fibers complex was constructed by aligning a nine-bp fragment of DNA of the pre-equilibrated RNA:fusomer (R1-F2) fibers onto the DNA fragment resolved in the PDB structure. The PDB’s were converted into Amber format, solvated, and ionized using the tLEaP package of AmberTools22(29).

*A/B-form analysis:* Structural propensity of the fusomer fibers toward an A-form or B-form geometry was evaluated by calculating the average rise per base pair in defined regions of the fusomer fibers. Reference values of 0.26 nm and 0.34 nm were used for canonical A-form RNA and B-form DNA, respectively. Each fusomer was partitioned into four subdomains: two segments between the KL and the nicked junction, and two segments spanning from the nick to the KL on the opposite strand. For each subdomain, rise per bp was computed by determining the center-of-mass (COM) distance between consecutive base pair helical centers and averaging over the length of the segment.

*Immunorecognition assessment*

*Primary human peripheral blood mononuclear cells (PBMCs) for analysis of cytokine secretion:* The blood of three healthy donors was collected, and its coagulation was prevented with lithium-heparin. The anticoagulated blood was mixed 1:1 with PBS at RT and layered on top of Ficoll-Paque. Blood samples were centrifuged at 900 X G with low acceleration and no brake for 30 minutes at RT. The monolayer containing the PBMCs was collected, and 1X HBSS was added at three times the volume. The solution was centrifuged at 400 X G for 10 minutes at RT. The washing step was repeated, and the mononuclear cells were resuspended in complete RPMI. Cells were then stained using AOPI for cell counting and plated at 2 X 10^5^ cells per well in a volume of 160 µL in a U-bottom plate.

To test the DNA cubes, RNA cubes, RNA fibers, and fusomer fibers in PBMCs, using Lipofectamine 2000 (L2K, Thermo Fisher Scientific) as a carrier, a final concentration of 10 nM of NANPs per well was added after being incubated with L2K (0.375 µL per well) at RT for 30 minutes and then was transfected in duplicate per donor. Samples were diluted in Opti-MEM to bring the volume of each well to 200 µL per well. Positive controls added to PBMCs included LPS (final 20 ng/mL), ODN2216 (final 5 µg/mL), and PHA-M (final 10 µg/mL). As a negative control, 1X PBS was added to PBMCs.

The supernatants were tested using a 15-multiplex plate (Quansys) following the supplier’s protocol. A Quansys ImagePro reader equipped with Q-view software was used to read the multiplex assays wherein a cytokine elevation of two-fold or more above the baseline was considered physiologically relevant.

*Reporter Cell Lines:* The following immune reporter cell lines were employed: THP-1 Dual, HEK-Lucia RIG-I, HEK-Blue hTLR3, HEK-Blue hTLR7, and HEK-Blue hTLR9 (InvivoGen). Cells were maintained at standard conditions of 37°C and 5% CO_2_, following InvivoGen’s protocols. THP1-Dual cells were plated at 1 X 10^5^ cells per well in a 96-well Greiner plate. HEK-Lucia RIG-I, HEK-Blue hTLR3, and HEK-Blue hTLR7 were seeded at a density of 5 X 10^4^ cells per well in a 96-well Greiner plate. HEK-Blue hTLR9 cells were seeded at 8 X 10^4^ cells per well in a 96-well Greiner plate. Immediately after plating, the cells were transfected with positive controls or a 10 nM final concentration of the RNA fibers or fusomer fibers. Prior to transfection, RNA fibers or fusomer fibers samples and positive controls (RNA cubes and 2′,3′-cGAMP) were incubated with L2K at RT for 30 minutes. For THP1-Dual cells, 1.9 μg/mL 2′,3′-cGAMP and 3 μg/mL Pam3CSK4 were used as positive controls to activate IRF and NF-κB signaling pathways, respectively. A final concentration of 10 nM per well of RNA cube was used as a positive control for HEK-Lucia RIG-I. For HEK-Blue hTLR3 and HEK-Blue hTLR9, 20 μg/mL of poly I:C was used as positive control. For HEK-Blue hTLR7, 5 μg/mL R848 was used as the positive control. Post-transfection, the cells were incubated at 37°C and 5% CO_2_ for ~24 hr, then assessed using immune reporter and cell viability assays. A QUANTI-Blue (InvivoGen) assay was used to assess secreted embryonic alkaline phosphatase (SEAP) levels in HEK-Blue hTLR3, HEK-Blue hTLR7, HEK-Blue hTLR9, and THP1-Dual cells, while a QUANTI-Luc (InvivoGen) assay was used to assess IRF activation in THP1-Dual and HEK-Lucia RIG-I cells according to the manufacturer’s guidelines. A Tecan Spark microplate reader was then used to measure absorbance at 638 nm for the QUANTI-Blue assay and luminescence (100 ms reading time) for the QUANTI-Luc assay. To assess cell viability post-transfection, MTS colorimetric assays (Promega) were performed according to the manufacturer’s protocol. The absorbance was read at 490 nm using a Tecan Spark microplate reader. Each experiment had 3 biological repeats, and the averages were normalized to untreated cells to obtain fold-induction or cell viability.

*cGAS-STING pathway mechanistic study:* A small molecule inhibitor, G140 (InvivoGen), was used at a final concentration of 10 nM to block cGAS-mediated activation of IRF in THP1-Dual cells. Cells were pretreated with G140 for 3 hours, followed by treatment with F1-30:F2-30 fusomer fibers and incubation for an additional 24 hours. After this period, IRF activation was quantified using the QUANTI-Luc™ 4 Lucia/Gaussia assay, and cell viability was assessed in parallel via MTS assay.

*Cellular uptake mechanistic study:* MDA-MB-231 cells were cultured and maintained at 37°C in a humidified incubator with 5% CO₂. Cells were seeded in Greiner 24-well plates at a density of 1.5 X 10⁵ cells per well in 800 µL of complete culture medium and allowed to adhere and grow for 48 hours to reach approximately 90% confluency. Cells were transfected with fusomer fibers labelled with Alexa Fluor 488 (AF488) at a final concentration of 100 nM, complexed with L2K as a carrier. Fusomer fibers-L2K complexes were prepared at room temperature for 30 min before transfection. Transfections were performed using a 200 µL transfection volume per well, bringing the final volume to 1 mL. Two temperature conditions were tested, where one plate was placed at 4°C and the other at 37°C. Cells were incubated for 24 hours post-transfection before flow cytometry analysis. For flow cytometry preparation, cell culture supernatants were collected into 1.5 mL microcentrifuge tubes. Adherent cells were detached using 0.25% Trypsin-EDTA, resuspended, and combined with the corresponding supernatants. Cells were pelleted by centrifugation at 700 X G for 5 minutes, after which the supernatant was carefully aspirated without disturbing the pellet. Cell pellets were resuspended in 1X phosphate-buffered saline (1X PBS) supplemented with 2 mM EDTA and 1% BSA. Flow cytometry was performed using an Attune NxT flow cytometer to assess fusomer fibers uptake, quantified as the percentage of AF488-positive cells. Data analysis was conducted using Attune Software, using the Overton subtraction method for population analysis. Histograms were displayed as relative fluorescence intensity.

*Fusomer fibers uptake assessment in 3D organ-on-a-chip*

*OrganoPlate Culture:* OrganoReady® Colon Caco-2 plates (Mimetas BV, The Netherlands) were prepared and used to assess uptake of fluorescently labelled fibers. The plate contains 64 chips made of Caco-2 tubules seeded against collagen I(30-33). The tubules form a leak-tight barrier that is used to assess the integrity of the barrier of the 3D culture(34). To prepare the Caco-2 OrganoPlate®, the gel channel was seeded with collagen-I and incubated overnight. Then, Caco-2 cells were seeded into the right channel inlet and incubated to attach to the gel matrix prior to the addition of media to all right and left channels. The plate was then placed on the OrganoFlow® rocker at 14°/8 min settings in a 37°C 5% CO_2_ incubator, and the Caco-2 cells form a 3D tubule in the right channel upon perfusion IRD 800 labelled RNA fibers and fusomer fibers were assembled, characterized, and were complexed with DOTAP DOPE and L2K (Thermo Fisher Scientific, USA) for 30 min at RT, before adding media to reach the final volume. The final concentrations tested were 20 nM, 50 nM, and 100 nM. Vehicle controls included DOTAP DOPE and L2K, and the negative control was untreated cells. All samples were tested in triplicate. Prior to transfection, all inlets and outlets were aspirated. Then, 50 µL of fresh media was added to all left inlets and outlets, while 50 µL of treatments were added to all right inlets and outlets. The OrganoPlate® was then incubated for 24 hours on a OrganoFlow® rocker (Mimetas BV, The Netherlands) at 14°/8 min settings in a 37°C, 5% CO_2_ incubator.

*TEER Analysis:* To measure the barrier integrity of the Caco-2 tubules, the transepithelial electrical resistance (TEER) values were measured. TEER values provide sensitive measurements of the tightness of the tubules, and insights about the toxicity of the transfected fibers by assessing their barrier induced disruption at the end of the experiment. Hence, TEER values were measured before transfection and 24 hours post transfection via OrganoTEER® (Ω*cm^2^) (Mimetas BV, The Netherlands). In this experiment, the transfected fibers were tested for their barrier disruption effects on the tubules. As a positive control for barrier disruption, a protein kinase inhibitor (staurosporine (33 nM)) was used as it significantly disrupts barrier integrity. Before measuring the TEER values, the OrganoPlate® was equilibrated to RT for 30 minutes then the TEER was measured at the designated timepoints.

*Imaging:* 24 hr after transfection, the experiment was terminated by fixing and staining the cells for imaging. The cells were washed and fixed for 15 min with 3.7% formaldehyde (Sigma) in HBSS with calcium and magnesium (Gibco). Then, the cells were washed twice with PBS (Gibco) for 5 min. Cells were stained immediately, the nucleus staining was performed using NucBlue™ fixed cell ReadyProbes™ reagent (Thermo Fisher, USA), following the manufacturer’s protocol. The OrganoPlate® was incubated for 15 min on a RT rocker. After incubation, the cells were washed with PBS for 1 min, and all wells were aspirated. 50 μL of PBS was added to all inlets and outlets including the gel inlets and the observation windows. For imaging, an OrganoPlate® Cytation 5 multi-mode microplate reader (BioTek, USA) was used, DAPI and CY5 filters were used at 4X. Fiji software was used for image analysis and uptake quantification.

*Nanopore sensing*

*Nanopore fabrication:* We used high-stress silicon nitride (250 MPa SiN) membranes supported by a Si chip as substrates for nanopore fabrication, as previously described(35). Nanopore fabrication was carried out using a JEOL 2010F transmission electron microscope operating at 200 kV. Following fabrication, nanopore chips were cleaned in hot piranha (2:1 H_2_SO_4_ / H_2_O_2_) for 30 min, followed by hot deionized water and drying under nitrogen, immediately prior to each experiment. After cleaning, nanopore chips were assembled in a custom flow cell equipped with Ag/AgCl electrodes, and a quick-curing silicone elastomer was applied between the chip and the cell to seal the device and thereby reduce the noise by minimizing the chip capacitance. Sample was added to cis (grounded) electrode and positive or negative voltage was applied to the trans chamber. Ionic current through the nanopore was measured using either an Axopatch 200B amplifier digitized at 250 kHz sample rates or a Chimera VC100 amplifier (Chimera Instruments LLC)(36) digitized at 4.17 MHz sample rates. Data analysis was carried out using Pyth-Ion software (https://github.com/rhenley/Pyth-Ion/) for loading, low-pass filtering, and extracting event parameters. Igor Pro (Wavemetrics) was employed in plotting.

*Sample preparation:* Complex solutions of NF-ĸB1 protein (MyBioSource,USA) and fibers were made by gently pipetting protein and fibers solutions, and then incubating at RT for one hr. The complex was stored at 4^o^C until used.

*Inactivation of NF-κB with fusomers of dual funtion*

*PBMCs for cytokine secretion analysis:* In similar experiments to those described above, NF-κB downregulation was assessed following spiking of plates with LPS. The supernatants for the positive controls were pooled 1:1:1 to be run on the multiplex plate. 24 hours after transfecting the cells with a panel of functionalized fusomer fibers, 20 ng of LPS was added to all treatments. After spiking the plate with LPS, plates were incubated for 20 hr at 37^o^C and 5% CO_2_. Afterwards, the plate was spun at 400 X G for 5 min, and the supernatants were transferred to a new 96-well plate for analysis. The supernatants were assayed using a 15-multiplex plate (Quansys) following the manufacturer’s protocol.

*Immunofluorescence analysis for NF-κB activation:* RAW 264.7 macrophage-like cells were cultured in DMEM supplemented with 10% fetal bovine serum (FBS) on the slides. Cells were reverse-transfected with fusomer fibers of dual function using Lipofectamine RNAiMAX transfection reagent (incubated complexes for 6 to 24 hours). Following transfection, cells were treated with LPS (100 ng/mL) for an additional 6 to 24 hours. After treatment, cells were washed once with PBS and fixed with 4% paraformaldehyde for 15 min at RT. Thereafter, cells were permeabilized with 0.1% Triton X-100, 1% BSA for 15 minutes and the nonspecific binding sites were blocked by adding 4% BSA for 1 hourr, followed by 2X wash with wash buffer. Subsequently, cells were incubated with primary antibody against NF-κB p65 (NF-κB p65 (D14E12) XP® Rabbit mAb #8242, Cell Signaling), followed by three washes in PBS. The cells were incubated with FITC-labelled goat anti-rabbit secondary antibody for 1 hour. After 5 minutes, the nuclear staining was achieved with Hoechst 33342, and then cells were mounted on slides using 50% glycerol in PBS and fluorescence images were acquired using an EVOS fluorescence microscope (Thermo Fisher Scientific).

*RT-PCR analysis:* Total RNA was extracted using TRIzol™ Reagent (Thermo Fisher Scientific) from adherent cells according to the manufacturer's protocol. RNA purity and integrity were determined by spectrophotometry (NanoDrop™ 1000 Spectrophotometer, Thermo Fisher Scientific) and gel electrophoresis, respectively. Total RNA was reverse transcribed to cDNA using a high-capacity RNA-to-cDNA kit (Thermo Fisher Scientific). The expression levels of mRNA encoding COX2 and iNOS were quantified using SYBR green fluorescence (Thermo Fisher Scientific) and the StepOnePlus real-time PCR system (Thermo Fisher Scientific), using the default cycling conditions recommended by the manufacturer. Each sample was run in triplicate; ActB and Hprt gene expressions were used as endogenous controls. Fold change was calculated using the comparative CT method (2−ΔΔCT). The primer sequences for gene products of interest are described in **Table S1**.

*Fusomer fibers with AgNCs for antibacterial activity*

*Minimum Inhibitory Concentration (MIC)/Minimum Bactericidal Concentration (MBC) Determination:* *S. aureus* UAMS-1 was grown from single colonies in LB at 37°C overnight in a GeneMate Incubated Shaker Mini with 200 rpm constant shaking. Bacteria were diluted to 1 X 10^6^ cells per well in a sterile 96-well plate with a final volume of 100 μL. Fusomer fibers carrying AgNCs were added to the bacteria with a concentration gradient. A Tecan Spark microwell plate reader was used to obtain optical density measurements. Initial optical density at 600 nm measurements were taken before incubation at 37°C for 20 hours. After incubation, the optical density at 600 nm was taken again to evaluate the minimum inhibitory concentration (MIC). Cells alone, antibiotic, and concentrations at and above the MIC were plated for CFU. The following day, colonies were counted on each plate to find the minimum bactericidal concentration (MBC) with at least 99.9% bacteria cell death. This has been described in **Figure S15**. A total of three biological repeats were performed. GraphPad Prism 9 was used to calculate the MBC.

*Mammalian cell viability assays:* HEK 293-FT cells, grown in DMEM, 2 mM L-glutamine, 1% PenStrep, and 10% heat inactivated FBS, were plated in a 96-well plate at a cell density of 4 X 10^4^ cells/well. After plating, cells were incubated at 37°C, 5% CO_2_ for 24 hours before treatment. Fusomer fibers withAgNCs, C12 AgNCs, silver control, fusomer, C12 hairpin, and 1X assembly buffer were used for cell treatments, and media was added to bring the final volume to 100 μL per well. The cells were then incubated for 24 hr prior to the addition of 20 μL of CellTiter 96^®^ AQueous one solution cell proliferation assay (MTS) solution to each well. The plate was incubated for 75 minutes before measuring absorbance at 490 nm using a Tecan Spark microplate reader(2). A total of three biological repeats, with three technical repeats for each experiment, were performed. GraphPad Prism 9 was used for data analysis and plotting.

*Excitation-Emission Spectra Analysis:* Fusomer fibers with AgNCs and C12 AgNCs were synthesized at 10 μM. After overnight incubation, 100 μL of each sample was placed into a 96-well black-walled plate and an initial measurement was taken. The Tecan Spark plate reader measured the intensity at emission wavelengths of 350-700 nanometers after being excited with wavelengths of 400-850 nm, with a bandwidth of 5 nm and step size of 5 nm. The gain was manually set at 150. The data was plotted as a heat map using GraphPad Prism.

*NU fusomer fibers with anticoagulant activity*

*Prothrombin time activated partial thromboplastin time, and thrombin time assessment:* Coagulation parameters, including Prothrombin Time (PT), Activated Partial Thromboplastin Time (APTT), and Thrombin Time (TT), were measured using clinical-grade reagents and instrumentation. Commercial kits from HemosIL (Werfen) were employed as follows: PT (Cat. No. 002002950), APTT (Cat. No. 0020006800), and TT (Cat. No. 0009758515), all used in accordance with the manufacturer’s instructions. Peripheral blood was collected from 10 healthy adult donors under the FMUSP ethical protocol NP 1378/18. All participants provided written informed consent prior to sample collection. Blood was anticoagulated with 3.2% sodium citrate and processed within 2 hours. Platelet-poor plasma was prepared by centrifugation at 2500 X G for 10 min at RT, pooled, and used within 8 hours. For each condition, 50 µL of NU fusomer fibers (final concentration: 3 µM) was added to 450 µL of pooled human plasma in 1.5 mL microcentrifuge tubes. Samples were incubated at 37°C for 30 minutes. Coagulation was initiated by automated addition of the respective reagents on an ACL TOP 350 CTS analyzer (Instrumentation Laboratory – Werfen), and clotting times were recorded in seconds. Assays were performed in compliance with clinical laboratory standards (CLSI H21-A5; ISO 15189). All reagents were certified for clinical use, and instruments were calibrated according to manufacturer guidelines. While statistical differences in clotting time were observed between treated and control samples, the short duration and high precision of these assays (CV <5%) limit their interpretability as a direct measure of biological effect.

*Gene silencing*

*RNA GFP DS assembly:* The sense and antisense strands were mixed at an equimolar ratio with HyPure grade water. Once mixed, they were incubated at 95°C for 2 minutes. Subsequently, 5X assembly buffer (containing 10 mM Mg2+ and 250 mM K+) was added at 20% of the final volume. The sample was then allowed to equilibrate at RT for 20 min before being stored at 4°C.

*Sample preparation:* Prior to all transfections using MDA-MB-231 eGFP cells, the RNA GFP dicer substrates (DS) and GFP fusomer fibers were assembled at a 1 µM concentration. These constructs were then complexed with L2K and incubated for 30 minutes at RT before transfection. Subsequently, samples were brought up to 50 µl using media to maintain final concentrations of 10 nM or 50 nM, as appropriate.

*Transfection* a*nd Imaging:* MDA-MB-231 eGFP cells were grown and maintained with complete DMEM (consisting of DMEM with 4.5 g/L D-Glucose, L-glutamine, 10% heat-inactivated FBS, 100 µg/mL penicillin, and 100 µg/mL streptomycin) and incubated at 37°C, 5% CO_2_. MDA-MB-231 eGFP cells were seeded in a 24-well Greiner plate at ~4 X 10^4^ cells per well in a 200 µl volume. The cells were incubated for 24 hours at 37°C with 5% CO_2_ before transfection. After the initial incubation, the cells were transfected with GFP fusomer at final concentrations of 10 and 50 nM, as well as with control RNA GFP dicer substrate (DS) at the same concentrations. All treatments were performed in a total volume of 50 µl, bringing the final volume to 250 µl/well. The cells were incubated at 37°C with 5% CO_2_ for 72 hours post-transfection. After treatment, the media was removed from the cells, and 100 µl of 1X PBS was added. The cells were then visualized using the EVOS cell imaging system.

**Supporting Figures**

**Figure S1**: Dehydration of fusomer fibers and RNA fibers. Schematic outline of the dehydration of constructs via lyophilization and vacuum drying, the different conditions of storage for a week, and rehydration and assessment of stability on native-PAGE. Native-PAGE showing the stability of the dehydrated samples via lyophilization and vacuum drying at 55°C as compared to the solution control stored at the same temperature, and the positive control stored at 4°C.

**Figure S2**: Nuclease sensitivity of fusomer and RNA fibers was assessed by incubation in 10% fetal bovine serum (FBS) at 37°C. Control samples consisted of fibers incubated in assembly buffer (89 mM Tris-borate, pH 8.2, 2 mM MgCl₂, and 50 mM KCl).

**Figure S3**: Immune recognition and cell viability following treatment with fusomer fibers and RNA fibers. (**a**) Type I and Type III interferon (IFN) production in PBMCs isolated from freshly collected blood of three healthy donors after treatment with DNA cubes, RNA cubes, RNA fibers (R1-15:R2-15), RNA-fusomer fibers (R1-15:F2-15), and fusomer fibers (F1-15:F2-15), with ODN 2216 as a positive control (mean ± SEM, n = 3). (**b**) Immune recognition of several fiber formulations in reporter cell lines HEK-Blue hTLR3, HEK-Blue hTLR7, HEK-Blue hTLR9, HEK-Lucia RIG-I, and THP1-Dual. Appropriate positive controls were included in each assay to confirm activation of NF-κB or IRF signaling pathways. (c) Cell viability of the reporter cell lines following 24-hour treatment with the RNA fibers and fusomer fibers panel (mean ± SEM, n = 3).

**Figure S4**: Mechanistic study of cGAS-mediated IRF activation. (**a**) Assessment of IRF activation after blocking cGAS-mediated activation of IRF pathway in THP1-Dual cells by G140. Cells were pretreated with G140 for 3 hours, followed by treatment with F1-30:F2-30 fusomer fibers and incubation for an additional 24 hours. Then, IRF activation was quantified using the QUANTI-Luc™ 4 Lucia/Gaussia assay, and (**b**) cell viability was assessed in parallel via MTS assay. Data shown as mean ± SEM, n = 3.

**Figure S5**: Flow cytometry analysis of A488-fusomer fibers uptake in MDA-MB-231 cells at (**a**) 37°C and (**b**) 4°C.

**Figure S6**: Characterization of different fibers with nanopores. (**a**) and (**b**), show a scatter plot of fractional current blockade and dwell time, and exponential fitting of the histogram of interevent time to calculate capture rate respectively for RNA fiber, (**c**) and (**d**) that for fusomer fibers with NF-kB decoy, (**e**) and (**f**) that for fusomer fibers of dual function, respectively. In (**e**), arrows show the additional population resulting from the DS RNA branching.

**Figure S7**: Cytokine production upon PBMCs treatment with different fusomer fibers (mean ± SEM, n = 3).

**Figure S8**: Immunofluorescence analysis for detection of NF-κB. (**a**) Schematic representation of two independent experiments of RAW 264.7 cells transfected with samples 1–4 prior to LPS stimulation. (**b**) Representative immunofluorescence images from two independent experiments showing nuclear staining (Hoechst) and NF-κB localization. (**c**) Quantification of NF-κB nuclear intensity.

**Figure S9**: Expression levels of Cox-2 (**a**) and iNOS (**b**) in RAW 264.7 macrophages. Cells were treated with complexes 1–4 (50 nM) for 24 hr, followed by stimulation with LPS (100 ng/mL) for an additional 6 hr. Gene expression was quantified by RT-qPCR, normalized to Actb and Hprt1 reference genes, and analyzed using the ΔΔCt method. Data is representative of one independent experiment. Treatments: (1) fusomer fibers, (2) fusomer fibers with NF-κB decoy, (3) fusomer fibers of dual-function, (4) NF-κB decoy duplex, and control (LPS only). On the left side of each panel, Cox-2 and iNOS expression levels in untreated and LPS-stimulated RAW 264.7 cells are shown.

**Figure S10**: Qualitative analysis of GFP downregulation using fusomer fibers functionalized with Dicer-substrate (DS) RNAs targeting GFP and complexed with L2K, as well as free DS RNA targeting GFP, similarly complexed with L2K, both at a final concentration of 10 nM. Untreated cells were included as a control. Each image panel shows two fields of view, brightfield and GFP fluorescence, acquired using a 10× objective.

**Figure S11:** Conformational analysis of various fibers and NF-kB fusomer from all-atom MD simulations. (**a**) RMSF of the three representative fibers. Shaded regions denote hairpin loops that do not participate in base stacking or hydrogen bonding and therefore show elevated fluctuations in comparison to the base-paired regions of the constructs. (**b**) RMSF of NF-κB in the bound and unbound states. In the unbound state, NF-κB exhibits increased fluctuations due to a clamp-like motion. Binding to the fusomer fiber suppresses this motion, reducing RMSF values below 2 Å. (**c**) RMSF of the fusomer fiber bound to NF-kB. For panel a, RMSF was calculated after aligning, at each frame, the RNA/DNA fiber to its initial coordinates. For panels (**b**) and (**c**), RMSF was obtained by separately aligning the protein and the fusomer fiber, respectively, to their initial coordinates (the crystal structure for NF-κB) prior to each RMSF calculation. (**d**) Number of hydrogen bonds between NF-κB and DNA as a function of simulation time, computed using a donor-acceptor distance cutoff of 3.5 Å and a donor-hydrogen-acceptor angle cutoff of 30°.

**Figure S12**: Monomer:protein complex is required for observing deeper current spikes: (**a**) NF-kB decoy duplex monomer, (**b**) current traces for NF-kB protein, and (**c**) complex of NF-kB protein and NF-kB decoy duplex monomer respectively at 400 mV in 1M KCl, 10 mM HEPES, 2 mM MgCl_2_ at pH 7.5. Used pore was 4.5-6.5 nm in size. Current traces were recorded at a sampling rate of 4167 kHz and low-pass filtered at 250 kHz.

**Figure S13**: Observation of NF-kB protein binding to fusomer fiber with NF-kB decoy: (**a**) current trace of fusomer fiber with NF-kB decoy, (**b**) NF-kB protein, and (**c**), and NF-kB protein and fusomer fiber with NF-kB decoy complex respectively at 200 mV with 8 nm pore in in 1M KCl, 10 mM HEPES, 2 mM MgCl_2_ at pH 7.5. Complex was prepared by mixing the protein and fiber in 2:1 ratio. The current signal was recorded for 40 s for each sample. Current traces were recorded at a sampling rate of 4167 kHz and low-pass filtered at 250 kHz.

**Figure S14**: Observation of NF-kB protein binding to fusomer fibers of dual function: (**a**) current trace of fusomer fibers of dual function,(**b**) 20 % NF-kB protein and 80% fusomer fibers of dual function, and (**c**) 30 % NF-kB protein and 70% fusomer fibers of dual function respectively at 150 mV with 12 nm pore in in 0.4M KCl, 10 mM HEPES, 2 mM MgCl_2_ at pH 7.5. 33.3 nM protein was used in b and c. The current signal was recorded for 10 min for each sample. Current traces were recorded at a sampling rate of 4167 kHz and low-pass filtered at 250 kHz.

**Figure S15**: Experimental workflow used to determine the minimum inhibitory concentration (MIC) and minimum bactericidal concentration (MBC) of AgNCs against UAMS-1 S. aureus. Bacteria were plated at 1 X 10^6^ cells per well and treated with a concentration gradient of AgNCs. After a 20 hr incubation at 37°C, the plate was evaluated for bacterial growth. The concentration that had no growth determined by taking the absorbance at 600 nm was found to be the MIC. The MIC and higher was diluted and plated to find the number of colony forming units (CFU) per mL. The sample with 99.9% bacteria death was determined to be the MBC.

**Figure S16**: Antithrombin activity of free NU172 aptamer at different concentrations. (**a**) Thrombin Time (TT), (**b**) Activated Partial Thromboplastin Time (APTT), and (**c**) Prothrombin Time (PT) tests with human plasma. Data are presented as the mean of at least two independent experiments, each with a minimum of six technical replicates. Statistical significance was assessed using the Kruskal–Wallis test followed by Dunn’s post hoc test compared with the control group. (*) p<0.05; (**) p<0.005; (***) p<0.0001 and (****) p<0.00001. Measurements exceeding 300 s were set to 300 s.

**Figure S17**: AFM-based analysis of fusomer fibers flexibility. Histograms show the distributions of flexibility values for each functionalized fusomer fiber: Fusomer fibers (F1-15:F2-15), Fusomer fibers of dual function, Fusomer fibers of dual function bound to NF-kB protein, Fusomer fibers with C12 Hairpin.

**Supporting Tables**

**Table S1**: Murine primer sequences for Quantitative Polymerase Chain Reaction (qPCR).

| *Cox2 F* | 5’-ATGGTGAAAACTGTACTACACCTG-3’ |
| --- | --- |
| *Cox2 R* | 5’-AACTTCGCAGGAAGGGGATG-3’ |
| *iNos F* | 5’-CAGGAACCTACCAGCTCACTCT-3’ |
| *iNos R* | 5’-ATGTGCTGAAACATTTCCTGTG-3’ |
| *Actb F* | 5’-CCTTCTTGGGTATGGAATCCTGT-3’ |
| *Actb R* | 5’-CACTGTGTTGGCATAGAGGTCTTTAC-3’ |
| *Hprt F* | 5’-TGACACTGGTAAAACAATGCA-3’ |
| *Hprt R* | 5’-GGTCCTTTTCACCAGCAAGCT-3’ |

F (Forward), R (Reverse).

**Table S2**: Parameters of Gaussian Fits for distributions shown in **Figure 2.**

|  | First peak, max | Second peak, max | Third peak, max | A_1_ / A_2_ | A_1_ / A_3_ |
| --- | --- | --- | --- | --- | --- |
| R1-15:R2-15 | 38 | 97 | - | 3.09 | - |
| F1-15:F2-15 | 50 | 95 | 138 | 2.52 | 1.67 |
| F1-8:F2-15 | 34 | 81 | - | 0.54 | - |
| R1-15:F2-15 | 37 | 96 | - | 1.78 | - |
| F1-15:F2-30 | 46 | 115 | - | 2.73 | - |
| F1-8:F2-30 | 36 | 92 | - | 3.00 | - |
| F1-15:R2-15 | 35 | 71 | 109 | 2.83 | 1.83 |
| F1-30:F2-15 | 44 | 125 | - | - | - |
| F1-30:F2-30 | 38 | 95 | - | 2.21 | - |

**Table S3**: Ratio of End-To-End distance and Contour Length obtained from AFM image analysis.

|  | Ree/Lc |
| --- | --- |
| R1-15:R2-15 | 0.861 |
| F1-15:F2-15 | 0.861 |
| F1-8:F2-15 | 0.817 |
| R1-15:F2-15 | 0.819 |
| F1-15:F2-30 | 0.821 |
| F1-8:F2-30 | 0.889 |
| F1-15:R2-15 | 0.843 |
| F1-30:F2-15 | 0.893 |
| F1-30:F2-30 | 0.850 |

**Table S4:** 2-way ANOVA summary. This is a summary of the statistical significance between different treatments shown in **Figure 3c** of the main text.

| Tukey's multiple  comparisons test | Predicted (LS) Mean diff | 95.00% CI of diff | Below  Threshold? | Summary | Adjusted  P Value |
| --- | --- | --- | --- | --- | --- |
| IFNa |  |  |  |  |  |
| NC vs. PC | -2885 | -3048 to -2723 | Yes | **** | <0.0001 |
| NC vs. DNA cube | -1402 | -1565 to -1240 | Yes | **** | <0.0001 |
| NC vs. RNA cube | -3652 | -3832 to -3471 | Yes | **** | <0.0001 |
| NC vs. R1-15:R2-15 fibers | -196.5 | -369.4 to -23.68 | Yes | * | 0.0149 |
| NC vs. F1-15:F2-15 fibers | -524.1 | -686.3 to -361.8 | Yes | **** | <0.0001 |
| NC vs. R1-15:F2-15 fibers | -167.4 | -334.4 to -0.3709 | Yes | * | 0.0491 |
| PC vs. DNA cube | 1483 | 1326 to 1640 | Yes | **** | <0.0001 |
| PC vs. RNA cube | -766.4 | -942.5 to -590.4 | Yes | **** | <0.0001 |
| PC vs. R1-15:R2-15 fibers | 2689 | 2520 to 2857 | Yes | **** | <0.0001 |
| PC vs. F1-15:F2-15 fibers | 2361 | 2204 to 2519 | Yes | **** | <0.0001 |
| PC vs. R1-15:F2-15 fibers | 2718 | 2556 to 2880 | Yes | **** | <0.0001 |
| DNA cube vs.  RNA cube | -2249 | -2425 to -2073 | Yes | **** | <0.0001 |
| DNA cube vs.  R1-15:R2-15 fibers | 1206 | 1037 to 1374 | Yes | **** | <0.0001 |
| DNA cube vs.  F1-15:F2-15 fibers | 878.3 | 720.8 to 1036 | Yes | **** | <0.0001 |
| DNA cube vs.  R1-15:F2-15 fibers | 1235 | 1073 to 1397 | Yes | **** | <0.0001 |
| RNA cube vs.  R1-15:R2-15 fibers | 3455 | 3269 to 3641 | Yes | **** | <0.0001 |
| RNA cube vs.  F1-15:F2-15 fibers | 3128 | 2952 to 3304 | Yes | **** | <0.0001 |
| RNA cube vs.  R1-15:F2-15 fibers | 3484 | 3304 to 3665 | Yes | **** | <0.0001 |
| R1-15:R2-15 fibers vs.  F1-15:F2-15 fibers | -327.5 | -495.8 to -159.2 | Yes | **** | <0.0001 |
| R1-15:R2-15 fibers vs.  R1-15:F2-15 fibers | 29.17 | -143.7 to 202.0 | No | ns | 0.9988 |
| F1-15:F2-15 fiber vs. R1-15:F2-15 fiber | 356.7 | 194.4 to 519.0 | Yes | **** | <0.0001 |
|  |  |  |  |  |  |
| IFNb |  |  |  |  |  |
| NC vs. PC | -99.91 | -268.2  to 68.41 | No | ns | 0.5668 |
| NC vs. DNA cube | -60.89 | -233.7  to 112.0 | No | ns | 0.9403 |
| NC vs. RNA cube | -353.8 | -526.7  to -181.0 | Yes | **** | <0.0001 |
| NC vs. R1-15:R2-15 fibers | -19.22 | -197.7  to 159.3 | No | ns | >0.9999 |
| NC vs. F1-15:F2-15 fibers | -41.7 | -214.6  to 131.2 | No | ns | 0.9911 |
| NC vs. R1-15:F2-15 fibers | -13.95 | -244.4  to 216.5 | No | ns | >0.9999 |
| PC vs. DNA cube | 39.02 | -123.3  to 201.3 | No | ns | 0.9912 |
| PC vs. RNA cube | -253.9 | -416.2  to -91.64 | Yes | *** | 0.0001 |
| PC vs. R1-15:R2-15 fibers | 80.69 | -87.63  to 249.0 | No | ns | 0.7826 |
| PC vs. F1-15:F2-15 fibers | 58.21 | -104.1  to 220.5 | No | ns | 0.9351 |
| PC vs. R1-15:F2-15 fibers | 85.96 | -136.7  to 308.6 | No | ns | 0.9096 |
| DNA cube vs.  RNA cube | -293 | -459.9  to -126.0 | Yes | **** | <0.0001 |
| DNA cube vs.  R1-15:R2-15 fibers | 41.67 | -131.2  to 214.5 | No | ns | 0.9911 |
| DNA cube vs.  F1-15:F2-15 fibers | 19.19 | -147.8  to 186.2 | No | ns | 0.9999 |
| DNA cube vs.  R1-15:F2-15 fibers | 46.94 | -179.2  to 273.1 | No | ns | 0.996 |
| RNA cube vs.  R1-15:R2-15 fibers | 334.6 | 161.8  to 507.5 | Yes | **** | <0.0001 |
| RNA cube vs.   F1-15:F2-15 fibers | 312.1 | 145.1  to 479.1 | Yes | **** | <0.0001 |
| RNA cube vs.  R1-15:F2-15 fibers | 339.9 | 113.8  to 566.0 | Yes | *** | 0.0003 |
| R1-15:R2-15 fibers vs.   F1-15:F2-15 fibers | -22.48 | -195.3  to 150.4 | No | ns | 0.9997 |
| R1-15:R2-15 fibers vs. R1-15:F2-15 fibers | 5.273 | -225.2  to 235.8 | No | ns | >0.9999 |
| F1-15:F2-15 fibers vs. R1-15:F2-15 fibers | 27.75 | -198.4  to 253.9 | No | ns | 0.9998 |
|  |  |  |  |  |  |
| IFN lambda |  |  |  |  |  |
| NC  vs. PC | -226.3 | -394.6  to -58.01 | Yes | ** | 0.0018 |
| NC vs. DNA cube | -140.2 | -308.5  to 28.13 | No | ns | 0.1704 |
| NC vs. RNA cube | -515.7 | -694.2  to -337.1 | Yes | **** | <0.0001 |
| NC vs. R1-15:R2-15 fibers | -21.38 | -189.7  to 146.9 | No | ns | 0.9998 |
| NC vs. F1-15:F2-15 fibers | -50.3 | -218.6  to 118.0 | No | ns | 0.973 |
| NC vs. R1-15:F2-15 fibers | -20.27 | -198.8  to 158.3 | No | ns | 0.9999 |
| PC vs. DNA cube | 86.14 | -71.30  to 243.6 | No | ns | 0.659 |
| PC vs. RNA cube | -289.3 | -457.6  to -121.0 | Yes | **** | <0.0001 |
| PC vs. R1-15:R2-15 fibers | 204.9 | 47.50  to 362.4 | Yes | ** | 0.0028 |
| PC vs. F1-15:F2-15 fibers | 176 | 18.58  to 333.5 | Yes | * | 0.0178 |
| PC vs. R1-15:F2-15 fibers | 206.1 | 37.73  to 374.4 | Yes | ** | 0.0064 |
| DNA cube vs. RNA cube | -375.5 | -543.8  to -207.2 | Yes | **** | <0.0001 |
| DNA cube vs. R1-15:R2-15 fibers | 118.8 | -38.64  to 276.3 | No | ns | 0.2725 |
| DNA cube vs. F1-15:F2-15 fibers | 89.89 | -67.56  to 247.3 | No | ns | 0.6122 |
| DNA cube vs. R1-15:F2-15 fibers | 119.9 | -48.41  to 288.2 | No | ns | 0.3405 |
| RNA cube vs. R1-15:R2-15 fibers | 494.3 | 326.0  to 662.6 | Yes | **** | <0.0001 |
| RNA cube vs. F1-15:F2-15 fibers | 465.4 | 297.0  to 633.7 | Yes | **** | <0.0001 |
| RNA cube vs. R1-15:F2-15 fibers | 495.4 | 316.9  to 673.9 | Yes | **** | <0.0001 |
| R1-15:R2-15 fibers vs. F1-15:F2-15 fibers | -28.92 | -186.4  to 128.5 | No | ns | 0.998 |
| R1-15:R2-15 fibers vs. R1-15:F2-15 fibers | 1.105 | -167.2  to 169.4 | No | ns | >0.9999 |
| F1-15:F2-15 fibers vs. R1-15:F2-15 fibers | 30.02 | -138.3  to 198.3 | No | ns | 0.9983 |

**Supporting References**

1.	Gupta AK, Marshall N, Yourston L, Rolband L, Beasock D, Danai L, et al. Optical, structural, and biological properties of silver nanoclusters formed within the loop of a C-12 hairpin sequence. Nanoscale Adv. 2023;5(13):3500-11.

2.	Rolband L, Yourston L, Chandler M, Beasock D, Danai L, Kozlov S, et al. DNA-Templated Fluorescent Silver Nanoclusters Inhibit Bacterial Growth While Being Non-Toxic to Mammalian Cells. Molecules. 2021;26(13).

3.	Tran AN, Chandler M, Halman J, Beasock D, Fessler A, McKeough RQ, et al. Anhydrous Nucleic Acid Nanoparticles for Storage and Handling at Broad Range of Temperatures. Small. 2022;18(13).

4.	Lyubchenko YL, Gall AA, Shlyakhtenko LS. Visualization of DNA and protein-DNA complexes with atomic force microscopy. Methods Mol Biol. 2014;1117:367-84.

5.	Shlyakhtenko LS, Gall AA, Filonov A, Cerovac Z, Lushnikov A, Lyubchenko YL. Silatrane-based surface chemistry for immobilization of DNA, protein-DNA complexes and other biological materials. Ultramicroscopy. 2003;97(1-4):279-87.

6.	Lushnikov AJ, Avila YI, Afonin KA, Krasnoslobodtsev AV. Characterization of RNA Nanoparticles and Their Dynamic Properties Using Atomic Force Microscopy. Methods Mol Biol. 2023;2709:191-202.

7.	Sajja S, Chandler M, Fedorov D, Kasprzak WK, Lushnikov A, Viard M, et al. Dynamic Behavior of RNA Nanoparticles Analyzed by AFM on a Mica/Air Interface. Langmuir. 2018;34(49):15099-108.

8.	Schindelin J, Arganda-Carreras I, Frise E, Kaynig V, Longair M, Pietzsch T, et al. Fiji: an open-source platform for biological-image analysis. Nat Methods. 2012;9(7):676-82.

9.	Sulc P, Romano F, Ouldridge TE, Doye JP, Louis AA. A nucleotide-level coarse-grained model of RNA. J Chem Phys. 2014;140(23):235102.

10.	Matek C, Sulc P, Randisi F, Doye JP, Louis AA. Coarse-grained modelling of supercoiled RNA. J Chem Phys. 2015;143(24):243122.

11.	Bohlin J, Matthies M, Poppleton E, Procyk J, Mallya A, Yan H, et al. Design and simulation of DNA, RNA and hybrid protein-nucleic acid nanostructures with oxView. Nat Protoc. 2022;17(8):1762-88.

12.	Phillips JC, Hardy DJ, Maia JDC, Stone JE, Ribeiro JV, Bernardi RC, et al. Scalable molecular dynamics on CPU and GPU architectures with NAMD. J Chem Phys. 2020;153(4):044130.

13.	Ivani I, Dans PD, Noy A, Perez A, Faustino I, Hospital A, et al. Parmbsc1: a refined force field for DNA simulations. Nat Methods. 2016;13(1):55-8.

14.	Zgarbova M, Otyepka M, Sponer J, Mladek A, Banas P, Cheatham TE, 3rd, et al. Refinement of the Cornell et al. Nucleic Acids Force Field Based on Reference Quantum Chemical Calculations of Glycosidic Torsion Profiles. J Chem Theory Comput. 2011;7(9):2886-902.

15.	Maier JA, Martinez C, Kasavajhala K, Wickstrom L, Hauser KE, Simmerling C. ff14SB: Improving the Accuracy of Protein Side Chain and Backbone Parameters from ff99SB. J Chem Theory Comput. 2015;11(8):3696-713.

16.	Jorgensen WL, Chandrasekhar J, Madura JD, Impey RW, Klein ML. Comparison of simple potential functions for simulating liquid water. The Journal of Chemical Physics. 1983;79(2):926-35.

17.	Yoo JJ, Aksimentiev A. Improved Parametrization of Li<SUP>+</SUP>, Na<SUP>+</SUP>, K<SUP>+</SUP>, and Mg<SUP>2+</SUP> Ions for All-Atom Molecular Dynamics Simulations of Nucleic Acid Systems. J Phys Chem Lett. 2012;3(1):45-50.

18.	Miyamoto S, Kollman PA. Settle - an Analytical Version of the Shake and Rattle Algorithm for Rigid Water Models. J Comput Chem. 1992;13(8):952-62.

19.	Andersen HC. Rattle - a Velocity Version of the Shake Algorithm for Molecular-Dynamics Calculations. J Comput Phys. 1983;52(1):24-34.

20.	Darden T, York D, Pedersen L. Particle Mesh Ewald - an N.Log(N) Method for Ewald Sums in Large Systems. Journal of Chemical Physics. 1993;98(12):10089-92.

21.	Martyna GJ, Tobias DJ, Klein ML. Constant pressure molecular dynamics algorithms. The Journal of Chemical Physics. 1994;101(5):4177-89.

22.	Allen MP, Tildesley DJ. Computer Simulation of Liquids: Oxford University Press; 2017 23 Nov 2017.

23.	Payne MC, Teter MP, Allan DC, Arias TA, Joannopoulos JD. Iterative minimization techniques for ab initio total-energy calculations: molecular dynamics and conjugate gradients. Reviews of Modern Physics. 1992;64(4):1045-97.

24.	Humphrey W, Dalke A, Schulten K. VMD: Visual molecular dynamics. Journal of Molecular Graphics & Modelling. 1996;14(1):33-8.

25.	Gowers RJ, Linke, Max, Barnoud, Jonathan, Reddy, Tyler John Edward, Melo, Manuel N., Seyler, Sean L., Domanski, Jan, Dotson, David L., Buchoux, Sebastien, Kenney, Ian M., & Beckstein, Oliver MDAnalysis: A Python Package for the Rapid Analysis of Molecular Dynamics Simulations. . (2019).

26.	Maffeo C, Aksimentiev A. MrDNA: a multi-resolution model for predicting the structure and dynamics of DNA systems. Nucleic Acids Res. 2020;48(9):5135-46.

27.	Suma A, Poppleton E, Matthies M, Sulc P, Romano F, Louis AA, et al. TacoxDNA: A user-friendly web server for simulations of complex DNA structures, from single strands to origami. J Comput Chem. 2019;40(29):2586-95.

28.	Anandakrishnan R, Aguilar B, Onufriev AV. ++3.0: automating p

prediction and the preparation of biomolecular structures for atomistic molecular modeling and simulations. Nucleic Acids Res. 2012;40(W1):W537-W41.

29.	Case DA, Aktulga HM, Belfon K, Cerutti DS, Cisneros GA, Cruzeiro VWD, et al. The AmberTools. J Chem Inf Model. 2023;63(20):6183-91.

30.	Trietsch SJ, Naumovska E, Kurek D, Setyawati MC, Vormann MK, Wilschut KJ, et al. Membrane-free culture and real-time barrier integrity assessment of perfused intestinal epithelium tubes. Nat Commun. 2017;8(1):262.

31.	Weller A, Hansen MB, Marie R, Hundahl AC, Hempel C, Kempen PJ, et al. Quantifying the transport of biologics across intestinal barrier models in real-time by fluorescent imaging. Front Bioeng Biotechnol. 2022;10:965200.

32.	Kosim K, Schilt I, Lanz HL, Vulto P, Kurek D. Intestinal Epithelium Tubules on a Chip. Methods Mol Biol. 2022;2373:87-105.

33.	Morelli M, Cabezuelo Rodriguez M, Queiroz K. A high-throughput gut-on-chip platform to study the epithelial responses to enterotoxins. Sci Rep. 2024;14(1):5797.

34.	Beaurivage C, Naumovska E, Chang YX, Elstak ED, Nicolas A, Wouters H, et al. Development of a Gut-On-A-Chip Model for High Throughput Disease Modeling and Drug Discovery. Int J Mol Sci. 2019;20(22).

35.	Larkin J, Henley RY, Muthukumar M, Rosenstein JK, Wanunu M. High-Bandwidth Protein Analysis Using Solid-State Nanopores. Biophys J. 2014;106(3):696-704.

36.	Rosenstein JK, Wanunu M, Merchant CA, Drndic M, Shepard KL. Integrated nanopore sensing platform with sub-microsecond temporal resolution. Nature Methods. 2012;9(5):487-U112.
